# Intracellular construction of topology-controlled polypeptide nanostructures with diverse biological functions

**DOI:** 10.1038/s41467-017-01296-8

**Published:** 2017-11-02

**Authors:** Li-Li Li, Sheng-Lin Qiao, Wei-Jiao Liu, Yang Ma, Dong Wan, Jie Pan, Hao Wang

**Affiliations:** 1 CAS Center for Excellence in Nanoscience, CAS Key Laboratory for Biomedical Effects of Nanomaterials and Nanosafety, National Center for Nanoscience and Technology (NCNST), Beijing, 100190 P. R. China; 2 University of Chinese Academy of Sciences (UCAS), Beijing, 100049 P. R. China; 3 State Key Laboratory of Hollow Fiber Membrane Materials and Processes, School of Environmental and Chemical Engineering, Tianjin Polytechnic University, Tianjin, 300387 P. R. China

## Abstract

Topological structures of bio-architectonics and bio-interfaces play major roles in maintaining the normal functions of organs, tissues, extracellular matrix, and cells. In-depth understanding of natural self-assembly mechanisms and mimicking functional structures provide us opportunities to artificially control the natural assemblies and their biofunctions. Here, we report an intracellular enzyme-catalyzed polymerization approach for efficient synthesis of polypeptides and in situ construction of topology-controlled nanostructures. We reveal that the phase behavior and topological structure of polypeptides are encoded in monomeric peptide sequences. Next, we elucidate the relationship between polymerization dynamics and their temperature-dependent topological transition in biological conditions. Importantly, the linearly grown elastin-like polypeptides are biocompatible and aggregate into nanoparticles that exhibit significant molecular accumulation and retention effects. However, 3D gel-like structures with thermo-induced multi-directional traction interfere with cellular fates. These findings allow us to exploit new nanomaterials in living subjects for biomedical applications.

## Abstract (web summary)

The intracellular topology of a nanostructure plays a major role in its interactions with the cell and accordingly, its biological applications. Here, the authors design peptides that intracellularly polymerize into elastin-like polypeptides and assemble into various topologies, each of which exhibits a distinct set of biological functions.

## Introduction

The topological structures of nanomaterials or bio-architectonics greatly impact the biological performance of organs and tissues^1–3^. Previous studies reported that the artificial topological nanostructures altered how the cells interact with material surfaces, directed stem cell differentiation^4–6^, affected cell migration^2, 7^, or modulated endocytosis^8, 9^. In addition, the topology of a natural multimolecular structure, such as signal complexes^10^, DNA^11, 12^, or proteins^13^, defined target signaling pathway activation and managed the response of the cells. Therefore, the intracellular topology of a nanostructure plays a major role in its interactions with the cell and accordingly, its biological applications. In vitro fabricated nanostructures may change because of the complicated physiological environment^14^. To accurately evaluate the intracellular topological effect of the nanomaterials, an in situ construction approach should be developed.

Observations from nature have given insight as to how small molecules can be controllably manipulated to construct complex intracellular superstructures that with diverse topologies and biological functions. Previous works have reported the in situ construction of tailored artificial nanostructures from small molecules under the control of enzymes^15–18^. Enzyme, as the fundamental and ubiquitous catalyst in biological system, plays a crucial role in major life activities^19^. Due to the high specificity to their substrates, enzymes were widely utilized to regulate the assembly/disassembly process in a certain region for drug release^20, 21^, bioimaging^22, 23^, tissue engineering^24, 25^, et al. However, forming well-defined functional nanostructures from small building blocks in complex cytoplasm environments still faces challenge. In particular, the dynamic and thermodynamic behaviors of these components undergoing assembly processes via noncovalent interaction in cells are crucial for mechanistic understanding but are also seen as an arduous process.

Artificially and genetically encodable thermo-sensitive elastin-like polypeptides (ELPs) had been utilized for controllable formation of nanostructures in biomedicine^26, 27^. The elastic repeat peptide units can polymerize into ELPs with extensibility beyond natural elastin and are capable of undergoing an entropy-driven chain collapse process with temperature change^28–30^. In vitro-synthesized ELPs have been successfully applied in tissue microenvironments^29, 31–33^. However, polypeptide synthesis in cells with controlled nanostructures and enhanced bio-functions was rarely reported. In this paper, the transglutaminase (TGase) we used is enable to create a covalent bond between the amino group of lysine residue and carboxamide group of glutamine residue, which exhibits a high resistance to proteolysis^33, 34^. Thus, the TGase was used as an endogenous high-efficient catalyst^24, 35^ to polymerize ELPs and fabricate thermal-induced topological controllable nanomaterials in cells. Because of these properties, the enzyme-specific polymerization and sequent induced self-aggregation open a gate to spy upon the intracellular topological effect, further better understand the inherent topology of molecular/multimolecular interactions.

Here, we report an intracellular TGase-catalyzed polymerization process used for both the preparation of ELPs and in situ construction of topology-controlled nanostructures. Through rational design of the sequences, the polypeptides exhibit various physiochemical properties and phase transition behaviors, allowing us to build up a multi-dimensional approach to elucidate intracellular polymerization and the self-aggregation process. Based on this approach, various topological nanostructures are developed in situ in cytoplasm and found to exhibit variable biofunctions towards retention efficiency and cell cytotoxicity. Interestingly, we find that intracellular polymerization-induced self-aggregation exhibits a new behavior for molecular accumulation in tumor cells. Unlike extracellular ELPs that exhibit high biocompatibility, gel-like ELPs in cells shows significant cytotoxicity during polymerization and the self-aggregation process.

## Results

### TGase-catalyzed polymerization and the sequence-encoded behavior of polypeptides

**Fig. 1:**
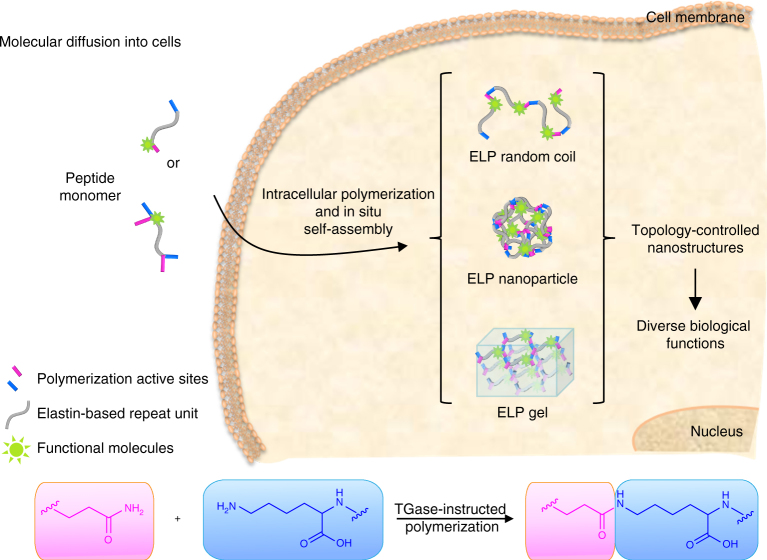
Schematic illustration of intracellular TGase-catalyzed polymerization and in situ controllable construction of nanostructures. The peptide monomer was composed of an elastin-based repeat unit, one-pair or two-pairs polymerization active sites, and a functional molecule. The TGase-instructed polymerization was performed via formation of isopeptide bond between the side chains of glutamine and lysine. Peptide monomers diffused into cells, and then polymerized intracellularly to form topology-controlled 1D elastin-like polypeptide (ELP) random coil, 3D ELP nanoparticle, and 3D ELP gel. These structure-differentiated ELPs exhibited diverse biological functions

By the de novo design of the monomeric peptide unit (Fig. 1), we control the topological growth and phase transition of the ELPs. The modular monomeric peptide is composed of a functional molecule (i.e., 4-(2-carboxypyrrolidin-1-yl)-7-(N,N-dimethylamino-sulphonyl)-2,1,3-benzoxadiazole (DBD), coumarin (CO), fluorescein isothiocyannate (FITC), cyanine 5.5 (Cy 5.5) or purpurin 18 (P_18_)), polymerization active sites (i.e., Q/K or QK/QK) and an elastin-based repeat unit (i.e., AVHPGVGP, HHPGVG, HDPGVG, HPGVGH, RLGVGFP, RLGVGLP, RLGVGDP, VHPGVG, VPHVG, and APGVG). The polymerization is catalyzed by TGase via the formation of the isopeptide bond between the glutamine and lysine side chain. The encoded sequence of polymerization active sites and elastin-based repeat unit regulated the upper critical solution temperature (UCST) and lower critical solution temperature (LCST) phase behavior, phase transition temperature, polymerization degree, and self-aggregated topological structure of the resulting polypeptides.

In the TGase family, TG2 is a widely distributed and expressed member, where the activity has been observed in diverse cellular physiological events^36, 37^. We first developed a TG2 polymerization approach in order to highly efficiently synthesize polypeptides for further intracellular application. We then designed 11 peptide monomers and labeled them with signal molecules, such as DBD, CO, P_18_, FITC, and Cy5 for further demonstration of the polymerization and self-aggregation (for the detailed synthesis, labeling, and characterization see Supplementary Methods; Supplementary Figs. 1–19). By optimizing the polymerization condition (Supplementary Table 1) in solution, we chose substrate-to-TG2 ratio (substrate: TG2 = 50 μM per U) in order to synthesize the target polypeptides. To demonstrate the successful synthesis of polypeptides, we designed CO-labeled and FITC-labeled monomeric units named CO-peptide 4 and FITC-peptide 4, respectively (Supplementary Fig. 20). The polymerization process was monitored by fluorescence resonance energy transfer (FRET) method. As a result, a significant FRET effect was observed upon introducing TG2 (0.5 U) to the mixture of CO-peptide 4 (5 μM) and FITC-peptide 4 (5 μM) in hydroxyethyl-piperazineethane-sulfonic acid buffer (HEPES buffer; Supplementary Figs. 21 and 22).

The synthesized peptide monomers were polymerized under the catalyzing of TG2 at 37 °C in HEPES buffer for 12 h. All the characterization results were listed in Table 1. The resultant linear topological polypeptides were measured by gel permeation chromatography (GPC) in order to determine the molecule weight (MW), degree of polymerization (DP), and polydispersity (PDI). Correspondingly, the polypeptide gels under a three-dimensional (3D) topological growth were described as cross-linking efficiency (CE%). The linear topological growth polypeptides were successfully polymerized with a MW around 15,000 ~ 30,000 Da and a DP about 17–36. Surprisingly, the relatively narrow PDI is an indication of the well-controlled polypeptide synthesis. Additionally, with high CE% over 89%, the polymeric network efficiently formed cross-linked 3D gel-like structures. To determine the sequence-encoded physiochemical behavior, we designed and synthesized a series of monomeric sequences with variable polymerization active sites and elastin-based repeat units listed in Table 1. The conservative repeated sequence in ELPs are inspired from natural elastic proteins, such as tropoelastin, collagen, and resilin. ELPs possess abundant Pro-residues and Gly-residues in a conservative repeated sequence (e.g., PGVG, GVGXP, and VHGP), which contribute to the temperature-triggered phase transitions behavior^30^. Recent reports pointed out that the Pro and Gly pairs which are spaced by other amino acids (red colored in Table 1) encode phase behavior of polypeptides^30^.

**Table 1:** Characterization of peptide monomers and their corresponding polymerized ELPs

Pep. No.	Sequence^a^	Pep. MW (D)	ELPs no.	ELPs MW^b^ (D)	PDI^c^ (Mw/Mn)	DP^d^	CE%^e^	Thermal behavior	Topological structure
C	AVHPGVGP	732.8	–	–	–	–	–	–	–
1	QHHPGVGK	858.5	**P1**	15,400	1.21	17–18	–	UCST	N/A^f^
2	QHDPGVGK	836.9	**P2**	17,900	1.28	21–22	–	UCST	N/A
3	QHPGVGHK	858.5	**P3**	15,500	1.21	18–19	–	UCST	N/A
4	QRLGVGFPK	1000.6	**P4**	28,000	1.25	27–28	–	UCST	Nanoparticle
5	QRLGVGLPK	966.6	**P5**	25,200	1.13	26–27	–	UCST	Nanoparticle
6	QRLGVGDPK	968.5	**P6**	25,500	1.17	26–27	–	UCST	Nanoparticle
7	QVHPGVGK	821.0	**P7**	29,700	1.29	35–36	–	LCST	Nanoparticle
8	QAPGVGK	754.9	**P8**	26,400	1.27	34–35	–	–	Random coil
9	QKVPHVGQK	1020.2	**P9**	–	–	–	90.6%	LCST	Gel^g^
10	QKAPGVGQK	912.2	**P10**	–	–	–	89.3%	–	Gel

^a^The conservative sequences in these elastin-repeated units included PGVG, GVGXP, and PHVG. The red colored amino acids inserted are significant in regulating the physicochemical properties of the synthesized ELPs

^b^The number average molecular weight of polypeptides was measured by gel permeation chromatography (GPC) equipped with refractive index detector and performed at 40 °C in DMF containing 0.4% LiBr with a flow rate of 1.0 ml min^−1^

^c^The polydispersity indexes (PDI) were calculated based on universal calibration method using polystyrene standards

^d^The degrees of polymerization (DP) were obtained by the dividing of the Mw of polypeptide to Mw of peptide monomeric unit

^e^The cross-linking efficiency (CE%) was defined as molecular percentage that was used in gel network cross-linking

^f^N/A means not available

^g^The gel with volume phase transition property

We concluded that: the increased charged residues (e.g., His or Arg) in the neighbor of conservative sequence displayed a lower MW after polymerization (such as **P1**–**P3**), which is attributed to the disfavored enzymatic activity during catalysis in the hydrophobic reaction cavity. The short length of the polypeptide chains dramatically affects their phase transition property and well-ordered thermo-induced assembly behavior^38–40^; The hydrophobicity of the guest residue *Xaa* (e.g., Phe > Leu > Asp) inserted between the conservative sequence (e.g., Gly-Val-Gly-*Xaa*-Pro) in **P4**–**P6** enhanced the transition temperature and sensitivity to temperature change (Supplementary Fig. 23). The ELPs based on these peptides sequences influence the topological structures during thermo-induced collapse process (detailed as below); The Ala next to the conservative sequence preferred to quench the phase transition behavior and then the formed ELPs (e.g., **P8** and **P10**) stabilized in a random coil conformation without sensitivity to temperature^30^; Arg/His rich cationic residues and the nearly zwitterionic character in both the tip of the conservative sequence overturned the traditional LCST behavior^41^ of ELPs into a typical UCST behavior (e.g., **P1**–**P6**). These results were verified by natural UCST resilin^30^. On the contrary, the non-polar residue-added sequence exhibited a constant LCST property of the resultant ELPs (e.g., **P7** and **P9**); topological growth into architectures of the polypeptides differed due to the designing of polymerization active sites with one or two reaction pairs (that is Gln/Lys or Gln-Lys/ Gln-Lys). The TG2-instructed polymerization enables the supply of a unified analysis platform in situ for the evaluation of topological structures self-assembled inside cells. The reaction sites were composed of cationic amino acid of Lys and polar amino acid of Gln, which were the strong modulators of hydropathy of ELPs. However, the formation of the isopeptide bond between the side chains of Gln and Lys neutralized Gln/Lys residues and weakened the hydropathy.

**Fig. 2:**
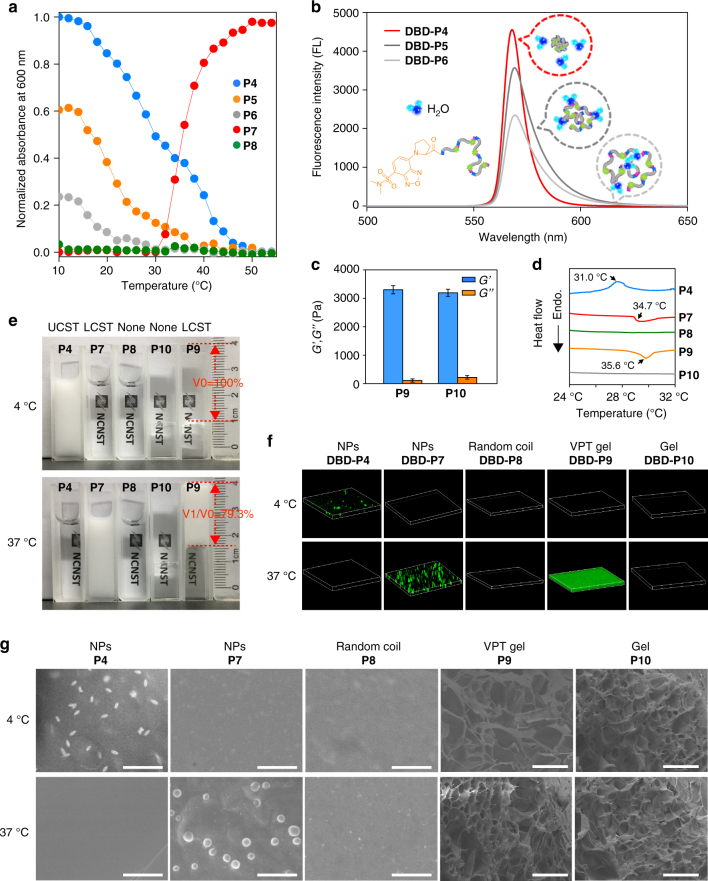
Characteristics of the synthesized polypeptides and the topological morphology. **a** Turbidity profiles of **P4**–**P8**. **P4**–**P6** were UCST-type ELPs, **P7** was a LCST-type ELP, and **P8** was a thermo-inert ELP. **b** Using of 4-(2-carboxypyrrolidin-1-yl)-7-(N, N-dimethylamino-sulphonyl)-2,1,3-benzoxadiazole (DBD) to monitor the collapse of **P4**–**P6** (200 μM). DBD-labeled ELPs were hydrated and fluorescence off at 37 °C, whereas turned on due to the dehydrate and collapse of the ELPs chains at 4 °C. **c** Rheological characterization of ELP gels (**P9** and **P10**). Average *G*ʹ and *G*ʺ values were recorded at a fixed frequency (0.5 Hz) and strain (5%) at 37 °C across a period of 70 min. Error bars indicate s.d. (*n* = 3) **d** Differential scanning calorimetry (DSC) thermograms for determining the phase transition temperatures. **e** Photographs of **P4** and **P7**–**P10** in aqueous solutions at 4 °C and 37 °C. **P9** volumes at 4 and 37 °C were indicated as *V*_0_ and *V*_1_, respectively. **f** Confocal images of DBD-labeled **P4** and **P7**–**P10** at 4 and 37 °C. ELP aggregation was monitored by the fluorescence changes of the polarity-sensitive probe DBD. NPs represented nanoparticles; VPT represented volume phase transition. **g** SEM images of **P4** and **P7**–**P10** at 4 and 37 °C. Scale bars, 4 μm for **P4**, **P7**, and **P8**; 50 μm for **P9** and **P10**

Next, for a detailed characterization of the polypeptides, we tested the phase transition behavior for these polypeptides under a linear topological growth (e.g., **P4**–**P8** in Fig. 2a, b) and a 3D topological growth (e.g., **P9** and **P10** in Fig. 2c). The temperature-dependent turbidimetry identified the LCST or UCST phase transition property of the linear growth polypeptides (Fig. 2a). Using an ultraviolet-visible spectrometer in HEPES buffer at a fixed polypeptide concentration (50 μM), the typical candidates of **P4** and **P7** for UCST and LCST were observed with a sharp soluble to insoluble transition during the temperature change. The longer polypeptide (**P7**) showed a sensitive phase transition in a narrow temperature range as reported in the literature^40, 42^. We aware that the hydrophobic amino acid neighboring to the Pro/Gly motifs contributed more to the phase transition induced collapse. The temperature-sensitive mechanism^43^ proved that the ELPs sequences in an extended structure have a hydration of amino linkages. For LCST, when the temperature increases, the weaken hydration leads to the hydrophobic interaction among the ELPs units. Thus, the more hydrophobic residues exhibited an improved collapsed property, by which the hydrophobic core formed accordingly with a stronger release capability of water molecules. By labeling with a polarity-sensitive DBD molecule^43^ (Supplementary Fig. 24), we introduced **P4**–**P6** to visualize this process. The quenched DBD motif on the chains of polypeptides by neighboring water molecules resulted in a light-up of fluorescence signals (Fig. 2b). Among three tested polymers with one amino acid residue variation (the guest residue: Phe, Leu, Asp in canonical Gly-Val-Gly-Xaa-Pro motif), the higher hydrophobic candidate **DBD-P4** in a collapsed state displayed the highest fluorescence enhancement of up to 7.5 folds compared to its extended structure (Supplementary Fig. 25). It was concluded that the hydrophobicity of elastic units contributed to the temperature sensitivity of nanoparticle assemblies. 3D topological growth of polypeptides with double-pair polymerization active sites successfully formed ELPs gels (e.g., **P9** and **P10**). We investigated the rheological property of these gels by dynamic time sweep rheological experiments (Fig. 2c; Supplementary Fig. 26). Larger *G*ʹ (storage modulus) than *G*ʺ (loss modulus) demonstrated the gel formation^41, 44^. The storage modulus and loss modulus of peptide 9 solution increased during the 70-min experimental period. The increase in *G*ʹ was faster than *G*ʺ, and within less than 52.6 min, *G*ʹ was equal to *G*ʺ indicating the transition from solution to gel. The data also indicated a final *G*ʹ value of ~ 3.30 kPa, signifying a structurally robust network that diverged from a physically assembled gel (with nominal *G*ʹ ranging from 10 to 10^2^ Pa^44^). The minimum gelation concentration of **P9** was 70 μM, which was determined by an inverted vial method at room temperature (Supplementary Fig. 27).

Then, in order to further investigate the intracellular polymerization and self-aggregation behavior, candidates of **P4** and **P7**–**P10** were collected and considered for the following reasons. Initially, we designed **P4**, which is a typical UCST phase transition ELPs with the transition temperature lower than 37 °C, in order to separately study the polymerization and self-aggregation process inside the cell through temperature regulation. Next, we carried out **P7** and **P8** with the same linear topological growth, which are respectively thermo-sensitive and thermo-inert ELPs, to monitor the simultaneous polymerization and self-aggregation process. Finally, we treated **P9** and **P10** with 3D topological growth, which were respectively thermo-induced volume phase transition and thermo-inert gel ELPs. We measured each phase transition temperature of these five candidates by differential scanning calorimetry (Fig. 2d). The transition temperature for the LCST group was lower than 37.0 °C (34.7 °C for **P7** and 35.6 °C for **P9**), providing an excellent model for interpreting the polymerization and accompanied self-aggregation in living cells. We endowed 31.0 °C transition temperatures for **P4** leading to a cooling adjusted collapse self-aggregation process. In contrast, the peptide monomers apparently exhibited no phase transition temperatures (Supplementary Fig. 28).

For visualization of the thermosensitivity and phase transition property of **P4** and **P7**–**P10** (Fig. 2e), we took photos of the ELPs in HEPES buffer solutions in cuvettes. In 37 and 4 °C atmospheres, the thermo-sensitive **P4**, **P7**, and **P9** exhibited turbidity conversions according to their phase transition temperatures. In comparative groups, **P8** and **P10** were kept in a transparent state under variable temperatures. Notably, the gel-like **P9** abruptly shrunk by about 20.7% volume. To determine whether the molecular environment plays a role in affecting the transition temperature and coacervation of ELPs, we presented various salts concentration (50–200 mM KCl) and protein contained solutions (5–20 mg ml^−1^ BSA and 150 mM KCl) for **P7** and **P9** (Supplementary Figs. 29 and 30). The results indicated that the higher salt concentration reduced the transition temperature of **P7**. However, in the 150 mM KCl solution (physical salt concentration) the transition temperature of **P7** was lower than 37 °C, which indicated that **P7** was in a collapsed state in cells at 37 °C. No significant change of the thermosensitivity and phase transition property of **P7** and **P9** were observed during the increase of concentration of BSA. We also characterized such a phase transition property of DBD-labeled **P4** and **P7**–**P10** with confocal images (Fig. 2f). The water sensitive probe (DBD) lit up the self-aggregated structures of the ELPs. The gel-like **P9** significantly turned on the fluorescence with consecutive green fluorescence. The linear ELPs of **P4** and **P7** in a collapsed state showed a dot-like distribution. The CD spectra of ELPs chains prior to the collapse were clearly characterized by a strong negative peak near 200 nm and a weak positive peak near 220 nm, which is indicative of the formation of random coil structures. Upon heating to 37 °C, the secondary structures of **P7** changed from a disordered state to a predominantly ordered state, where a pronounced reduction in the negative band at 200 nm^45^ occurred (Supplementary Fig. 31). Additionally, we statistically analyzed the diameters and size distribution of nanoparticles in a dried state by scanning electron microscope (SEM). According to SEM results, the size distributions were 658 ± 42 nm and 872 ± 190 nm for **P4** and **P7**, respectively (Supplementary Figs. 32 and 33). Both narrowed distribution of the aggregated nanoparticles might attribute to the small PDI (<1.3 in Table 1) of the ELPs during polymerization. For this, TG2-catalyzed polymerization and simultaneous nanoaggregation contributed to a novel platform for construction controllable nanostructures inside cells. The temperature-dependent SEM images of **P4** and **P7**–**P10** then revealed that the nanostructures can be well controlled by temperature change (Fig. 2g). The typical temperature-induced self-aggregation process for **P4** revealed that the nanoparticles were in a temperature-dependent growth form (Supplementary Fig. 34). Also, the lyophilized hydrogel in the swelling state of **P9** and **P10** at 4 °C exhibited larger pores, while the hydrogel of **P9** with volume phase transition displayed networks with 2–3 times smaller pores within tens of microns at 37 °C. On the contrary, the thermo-inert gel of **P10** exhibited no significant change. Taken together, the morphologies of nanostructures were demonstrated by using SEM (Fig. 2g), and the nanoparticle size, size distributions were investigated by DLS (Supplementary Fig. 35). The linear ELPs (e.g., **P4** and **P7**) collapsed into nanoparticles and the gel ones clearly displayed network architectures (e.g., **P9** and P**10**). Otherwise, the random coil ELPs (**P8**) showed no obvious nanostructure. Surprisingly, the shapes of the nanoparticles were distinct, while the reason right now cannot be explained.

### Endogenous TG2 polymerization and polypeptide intracellular self-aggregation

**Fig. 3:**
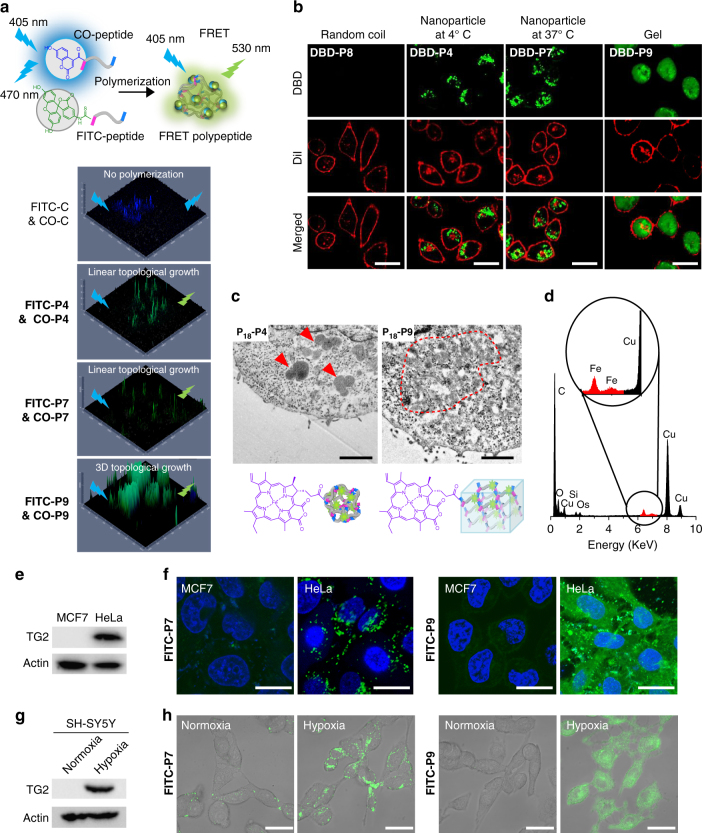
Intracellular polymerization and in situ aggregation of peptide monomers. **a** TG2-catalyzed intracellular polymerization of peptide monomers was monitored by fluorescent resonance energy transfer (FRET) technique. The 3D confocal images displayed the FRET effect of control, **P4**, **P7**, and **P9** inside HeLa cells at 12 h-post treatment. The feed ratio of FITC-peptide (fluorescein isothiocyannate-peptide) and CO-peptide (coumarin-peptide) was 1:1. **b** Confocal images displayed the nanostructures (green) formed from **P4** (at 4 °C) and (**P7**, **P9**) (at 37 °C). **P8**, **P4**, **P7**, and **P9** were ELP random coil, UCST-type ELP, LCST-type ELP, and ELP gel, respectively. Cell membrane, red. Scale bar, 30 μm. **c** The ultrathin cell sections of HeLa cells. Fe-chelated P_18_ was used to identify the formed nanostructures and for contrast enhancement. Red arrows indicated **P**_**18**_ **-P4** 3D nanoparticles; red dotted circle indicated **P**_**18**_ **-P9** gel. Scale bar, 1 μm. **d** Energy dispersive spectrometry (EDS) of the nanostructures formed from **P**_**18**_ **-P4**. The Fe element signal (red highlighted) made the **P**_**18**_ **-P4** from the biological background stand out. **e** MCF-7 and HeLa cells were lysed. TG2 protein was examined by western blotting. **f** Fluorescein isothiocyannate (FITC)-labeled **P7** and **P9** were incubated with MCF-7 or HeLa cells for fluorescence imaging. **FITC-P7** and **FITC-P9**, green; nucleus, blue. Scale bar, 30 μm. **g** The expression of TG2 protein in SH-SY5Y cells was modulated by normoxia (N) and hypoxia (H) conditions. **h** Peptide monomer polymerization and in situ aggregation was dependent on the expression of TG2. **FITC-P7** and **FITC-P9**, green. Scale bar, 50 μm

Introducing peptides to cells, we initially identified the internalization pathway during peptide monomers’ treatment (Supplementary Fig. 36). By inhibiting the endocytosis through 4 °C incubation, micropinocytosis by amiloride, clathrin-mediation by sucrose, and caveolae-mediation by β-cyclodextrin, we confirmed that the peptides were freely diffused into cells. For intracellular polypeptide polymerization, we added CO and FITC-labeled peptides (control and 4, 7, 9) as a FRET pair (1:1 molecular ratio with an original concentration of 300 μM) into TG2 overexpressed HeLa cells. By reconstructed 2D confocal images, we found that **P4**, **P7**, and **P9** exhibited a remarkable FRET process compared to that of the control (Fig. 3a). Moreover, we validated the reliability of the FRET signals in cells through a FRET response (FR) parameters^46^ for confirmation of polymerization-induced FRET instead of nonspecific intermolecular interactions. The statistical significances between positive HeLa cells and negative MCF-7 ones proved to us that FRET effect was originated from intracellular polymerization (Supplementary Fig. 37). On the contrary, no FRET effect was observed upon excitation at 405 nm for the control peptide without polymerization active sites. The linear ELPs of **P4** and **P7** at 37 °C found FRET fluorescence in cytoplasm with inhomogeneous distribution. In contrast, the gel **P9** expressed enhanced homogeneous fluorescence distribution intracellularly with the FR. Again, the intracellular polymerized ELPs were further isolated and characterized in the cell lysates. The FITC**-**labeled **P4** was obtained by thermoprecipitation method^47^ and characterized by GPC (Supplementary Fig. 38). In addition, the gel one, such as **P9**, was labeled with Fe^2+^ coordinated P_18_ were isolated from cells and confirmed by and SEM/EDS (Supplementary Fig. 39).

Besides intracellular polymerization, we also visualized the polymerization-induced self-aggregation process (Fig. 3b). The DBD-labeled thermo-sensitive ELPs of **P4**, **P7**, and **P9** presented green fluorescence signal in the collapsed state. Below the UCST for **P4** or up the LCST for **P7**, the linear ELPs chains collapsed and twisted with each other, subsequently the generated hydrophobic domains could include DBD signal molecules and significantly lit up dot-like fluorescence in cytoplasm. For the gel-like **P9**, the homogeneous network cross-linking occurred and distributed throughout the entire cytoplasm. To rule out the possibility of nonspecific interaction induced fluorescence enhancement, we monitored the **P4** self-aggregates inside the TG2 positive and negative cells under 37 and 4 °C conditions in parallelly. We obtained the negative results in the MCF-7 cells but positive results in HeLa cells (Supplementary Fig. 40). Also, the 10-fold enhancement of the fluorescence signal in the intensity profiling at 37 and 4 °C comprehensively demonstrated the thermo-induced self-aggregation property of ELPs intracellularly (Supplementary Fig. 41). Additionally, comparing to **P8** and **P10**, which were thermo-inert, the in situ self-aggregated **P7** and **P9** displayed a statistical difference with the aggregation induced fluorescence turned on capability (Supplementary Figs. 42 and 43).

To obtain the morphology of the nanoaggregates in cells, we carefully prepared the ultrathin sections of treated cells for TEM study (Fig. 3c). In this experiment, we labeled the peptide monomeric units (peptide control, 4 and 9) with Fe^2+^ coordinated P_18_ molecules to trace the nanoaggregates. Surprisingly, after polymerization and self-aggregation for 12 h in cells, the nanoparticles (red arrows) and gels (red dotted circles) were clearly visualized (Fig. 3c). Then, the nanostructures were confirmed by EDS with Fe element (Fig. 3d) and compared to the control (Supplementary Fig. 44). Statistically, the average size distribution of nanoparticles formed in cells was 770 ± 130 nm, which displayed a controllable morphology (Supplementary Fig. 45). To the best of our knowledge, this is the first example for constructing size-controllable nanoaggregates intracellularly. Meanwhile, the samples of gels were also observed in cytoplasm and confirmed by EDS (Supplementary Fig. 46).

In our system, the polymerization was specifically dependent on the endogenous TG2. By using western blot experiments, we confirmed that the TG2 were significantly overexpressed in HeLa cells than that in MCF-7 cells^48^ (Fig. 3e). Peptides 7 and 9 labeled with FITC were chosen to introduce into MCF-7 and HeLa cells, independently. As expected, we obtained the dot-like and continuous distributed green fluorescence in HeLa cells but much weaker fluorescence in MCF-7, implying that ELPs polymerization inside cells was dependent in TG2 expression (Fig. 3f). Given the fact that the different cells might cause variable peptide internalization, we further tested the polymerization in the same SH-SY5Y cells (Fig. 3g). The TG2 expression of SH-SY5Y cells was readily used to adjust the normoxia or hypoxia culture conditions of SH-SY5Y in order to obtain the various TG2 expressions in cells. Under the hypoxia condition, the SH-SY5Y cells will up-regulate expression of TG2^49^. Within the same experimental condition, the remarkable fluorescence signals were visualized in hypoxia SH-SY5Y cells (Fig. 3h). Based on the above results, we believe that polymerization was specifically catalyzed by endogenous TG2.

### The process of intracellular polymerization and self-aggregation

**Fig. 4:**
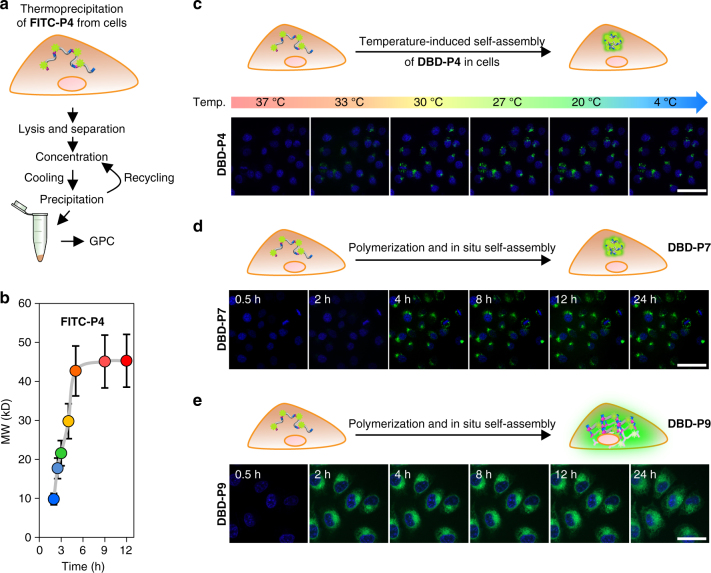
Intracellular polymerization dynamics of peptide monomers and aggregation process of ELPs. **a** Schematic illustration of thermoprecipitation of **FITC-P4** from cells. **b** Time-dependent intracellular growth of **P4**. The FITC-labeled **P4** was obtained at different time point by thermoprecipitation method. The molecular weights (MWs) of these **FITC-P4** were measured by GPC. Data were presented as mean ± s.d. (*n* = 5). **c** The thermodynamic self-assembly process of **DBD-P4** in HeLa cells during the cooling procedure. The cells were incubated with DBD-peptide 4 (600 μM) at 37 °C for 12 h before cooling down to 4 °C. **DBD-P4**, green; nucleus, blue. Scale bar, 55 μm. Real-time observation of **d**, linear growth and **e**, gel-like ELP formation and in situ aggregation. **DBD-P7** and **DBD-P9**, green; nucleus, blue. Scale bar, 50 μm

Understanding the chain growth and self-aggregation mechanism in a biological environment greatly helps us to rationally design functional superstructures. By using the real-time quantitative analysis method, we attempted to clarify the polymerization and synchronized self-aggregation processes and revealed their kinetic and/or thermodynamic behaviors inside cells (Fig. 4). In order to obtain the evidences of the intracellular polymerization, we undertook a phase bioseparation approach named thermoprecipitation^47^ to purify the resultant ELPs (e.g., **FITC-P4**), which was clearly illustrated in Fig. 4a. Based on the method above, we monitored the polymerization process from 2 to 12 h (Fig. 4b). The results implied that intracellular TG2 polymerization exhibited a time-dependent enzymatic catalysis characteristic. The MW of ELPs increased rapidly at the initial 0–6 h and reached a stable state over 6 h. The MW of the separated ELPs inside cells was similar to that obtained in buffers. The self-aggregated process of ELPs (e.g., **DBD-P4**) was then monitored with an isothermal cooling procedure. The temperature was cooled down from 37 to 4 °C within 5 min. As shown in Fig. 4c, the self-aggregation of nanoparticles was a fast-kinetic process and displayed a highly sensitive collapsed behavior in seconds below the transition temperature (e.g., 31.0 °C for **P4**). The nanoparticles were kept stable during the following cooling procedure from 30 to 4 °C. The results indicated to us that after the slow topological growth of the ELPs up to a certain MW, the phase transition induced aggregation formed instantly. The behavior of ELPs underwent a unimers-to-aggregate transition^29^ upon cooling lower than their transition temperatures. Finally, the above assumption was confirmed by time-lapsed confocal observation of **DBD-P7** (Fig. 4d) and **DBD-P9** (Fig. 4e). **DBD-P7** occurred in a spatial chain growth behavior in a random coil state (quenched DBD fluorescence) at the start of the 4 h. When the ELPs reached to about 30 kD, which was correlated to the results in Fig. 4b, the **DBD-P7** immediately collapsed into nanoparticles and turned on the DBD fluorescence at the onset of the 4 h and lasted up to 24 h. Interestingly, with the same mechanism, the 3D topological growth of the **DBD-P9** (Fig. 4e) in the first 2 h exhibited an irregular situation of quenched fluorescence. The network cross-linking began to hydrogelate and form the regular hydrophobic domain during 2–4 h, which showed a significant fluorescence lighted up process. The homogeneous fluorescence distribution lasted from 4 to 24 h. It is worth noting that the hydrogelation interaction and in situ volume phase transition of **DBD-P9** inside cells might influence the cell viability.

### The biological function affected by topological growth and phase transition property of ELPs

**Fig. 5:**
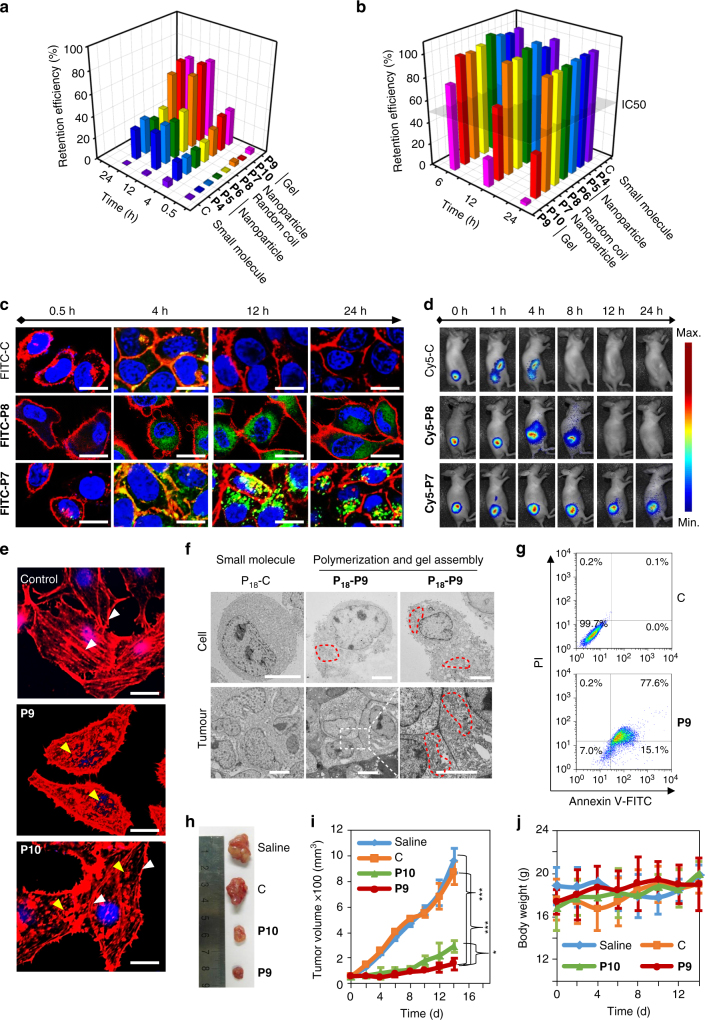
Biological functions of 1D and 3D ELP nanostructures. **a** Retention efficiencies of **P4**–**P10** and the non-polymerized peptide control were measured at 0.5, 4, 12, and 24 h post peptide monomer addition. The retention efficiency was defined as the percentage of cell trapped peptides to the total feeding peptides. **b** Cytotoxicity of **P4**–**P10** and the non-polymerized peptide control. Peptide monomers were fed at a concentration of 600 μΜ. **c** Fluorescence imaging of **FITC-C**, **FITC-P8**, and **FITC-P7** in HeLa cells at 0.5, 4, 12, and 24 h post treatment. **FITC-C**, **FITC-P8**, and **FITC-P7**, green; cell membrane, red; nucleus, blue. Scale bar, 30 μm. **d** Longitudinal fluorescence imaging of Cy 5-labeled peptide C, 7, and 8 treated mice that bearing HeLa tumor xenograft. All peptides were i.v. injected at a dose of 5.0 mg kg^−1^. The whole-body fluorescence images were monitored longitudinally using a Maestro fluorescence imager. **e** The fluorescence images of cell actin filaments (stained with Rhodamine Phalloidin) after treatment with peptide control, peptide 9 and 10 for 12 h. White and yellow arrows represented intact and disrupted actin filaments, respectively. Actin filament, red; nucleus, blue. Scale bar, 10 μm. **f** Bio-TEM images of ultrathin sections of HeLa cells and tumor tissues at 12 h post administration of peptide C and 9. Fe-chelated P_18_ was labeled on all peptides to enhance the imaging contrast. Red dotted circles represented the ELP gel nanostructure. Scale bar, 5 μM. **g** Apoptosis was analyzed in peptide C and peptide 9 treated HeLa cells by FACS. **h**, Representative images of HeLa xenograft tumors collected from the mice after treatment with saline, peptide c, peptide 9, and peptide 10 at day 14. Ruler unit, cm. **i** The HeLa tumor growth curves after i.v. injection of saline, peptide c, peptide 9, and peptide 10 at a dose of 5 mg kg^−1^. Error bars indicated s.d. (*n* = 3). The statistical significance (*p < 0.05 and ***p < 0.001) between all pairs of components was assessed using one-way analysis of variance (ANOVA) followed by post hoc Tukey’s test. **j** The body weight variation of HeLa tumor-bearing mice during treatment. Error bars indicated s.d. (*n* = 3)

To evaluate the biological functions of the various topological growths and collapsed ELPs (e.g., **P4**–**P10**) inside the cell, we systematically compared the retention efficiency and cell viability during intracellular polymerization and self-aggregation (Fig. 5a, b). By labeling peptides with FITC, we investigated the time-dependent retention efficiency, which was defined as the percentage of peptides blocked inside cells to the total feeding peptides in HeLa cells by a UV/vis spectrometer (Fig. 5a). Because they were small molecules (e.g., control peptide: C), almost all molecules were cleared out from the cell at 24 h resulting a retention efficiency of 0.2%. On the contrary, polymerization intracellularly (e.g., **P4**–**P10**) contributed to a significant enhancement of the retention efficiency over 28% and lasting up to 24 h. What’s more important was that the in situ self-aggregation of these ELPs (e.g., **P7** and **P9**) in cells further improved the retention efficiency due to the possibility of the intracellular protease degradation through tightly compacted nanostructures and enhanced hydrophobicity. This implied that enzyme-specific polymerization-induced self-aggregation might be the new approach to high-efficiency molecular accumulation. Furthermore, the time-dependent cell viabilities were carried out (Fig. 5b). Astonishingly, beyond a traditional consideration of the high biocompatibility for ELPs, the 3D topological polymerization inside the cells (e.g., **P9** and **P10**) dramatically induced cell death, while the thermo-sensitive ELPs gel (e.g., **P9**) accreted the cell death based on the simultaneously volume phase transition induced multidirectional forces (Fig. 5b; Supplementary Fig. 47). We also observed the ELPs gels exhibited a dose-dependent cell cytotoxicity behavior and a specific cell killing correlated with TG2 expression (Supplementary Figs. 48 and 49). However, the random coil and nanoparticles ELPs (e.g., **P4**–**P8**) are biocompatible for cells under the same concentration.

Based on the good biocompatibility of the linear topological growth ELPs and high retention efficiency, we utilized the typical candidate –**P7** as a demo for long-term retention of fluorescence molecules for bioimaging (Fig. 5c). In contrast to small molecules (**FITC-C**) and thermo-inert ELPs (**FITC-P8**), **FITC-P7** collapsed to form nanoparticles with green dots distributions, which significantly increased the molecule’s retention and prolonged the statistical fluorescence signal for up to 24 h. The in situ enhanced fluorescence intensity after nanoparticle formation were due to dramatically enhanced retention efficiency, which was benefit for bioimaging. For further in vivo demonstrations, we displayed the Cy5-labeled peptide C, 8 and 7 on the tumor-bearing mice models (Fig. 5d). As expected, the linear topological growth and aggregation of the nanoparticles of **Cy5-P7** exhibited the longest HeLa tumor bioimaging time up to 24 h.

The mechanism of intracellular 3D gel-induced cell death is not known. In order to gain a holistic understanding of this mechanism, we stained the actin filaments of the cells after treatment of the control peptide, peptide 9, and peptide 10. As compared to the well-defined long actin filaments of cells treated by the control peptide, the gel-like **P9** and **P10** exhibited fragmentary actin filaments, especially for **P9** (Fig. 5e). The phenomena could be explained that the gel formation prevents the diffusion and assembly of actin in cytoplasm and the volume phase transition further damaged the existing actin filaments. We then observed the morphology of gels formed inside cells and in tumor by TEM (Fig. 5f). Compared to the control, the **P**_**18**_ **-P9** formed inside cells or tumor exhibited a significant enhanced contrast (red dotted circle) during the hydrogelation. The flow cytometry results by Annexin V-FITC/PI apoptosis detection kit verified that intracellular 3D elastic ELPs gel formation would induce apoptosis (Fig. 5g). After the treatment of HeLa cells by peptide 9 for 12 h, about 15.1 % cells represented an early apoptosis and 77.6% were in late apoptosis. We also observed the apoptosis phenomenon after hydrogelation for 12 h in cells or tumors in ultrathin sections (Supplementary Figs. 50 and 51). We concluded that it was possible to use peptide-based small molecules to intracellular polymerize and self-organize into 3D nanostructures as drug-free agents for cancer therapy. To verify this hypothesis, we displayed this treatment approach on a tumor-bearing nude mice model with a dose of 5 mg kg^−1^ treatments by PBS, peptide C, 9 and 10 for two weeks. A therapeutic efficacy was observed according to the tumor size statistical analysis (Figs. 5h, i; Supplementary Fig. 52). Obviously tumor growth inhibitions were obtained based on the collected tumor tissues expectedly. To our surprise, with the volume phase transition property, a group of **P9** exhibited better antitumor activity than the group of **P10** after 2 weeks of treatment with a negligible body weight change (Fig. 5j).

## Discussion

In conclusion, we built an intracellular polymerization-induced self-aggregation nanosystem. By decoding variable peptide sequences, we controllably regulated the topological structures that exhibit unexpected biological functions. From our results, we conclude that small peptide units can be high-performance polymerized and self-aggregated into topologically controlled nanostructures, resulting in high efficiency of molecular accumulation and retention inside cell. By using the FRET effect, in situ cell lysate isolation of ELPs and aggregation induced fluorescence turn on molecules, we demonstrated that the TG2-catalyzed polymerization and self-aggregation did indeed occur intracellularly with a controllable manner, where polymerization was a slow chain growth process (in hours) and self-aggregation was a temperature-dependent fast process (in seconds). Correspondingly, TEM of the cells and tumor tissues clearly illustrated the controllable nanostructure inside cells and tumors. We therefore systemically analyzed the diverse biological functions of constructed materials in living subjects. According to our previous work, which displayed an assembly-induced retention effect, we found linear growth ELPs and their nanoparticles provided us biocompatible candidates with significantly enhanced retention efficiency for various biological applications such as high-performance bioimaging, sustainable drug release etc. Surprisingly, the 3D gel-like ELPs with volume phase transition property at 37 °C exhibit an accelerated cell apoptosis function, which can potentially be used for drug-free cancer therapy. We anticipate that these findings will inspire others to develop nanomaterials through an in vivo self-assembly approach with desirable biological functions.

## Methods

### TG2-catalyzed polymerization in solution

The TG2 was stored in HEPES buffer (50 mM HEPES, pH 7.4, 5 mM CaCl_2_, 10 mM dithiothreitol, 10% (W/V) glycerol). The peptide monomers were pre-dissolved in HEPES buffer with an original concentration of 10 mM. TG2-catalyzed polymerization was carried out at 37 °C for 12 h by mixing the TG2 and each peptide monomer stock solutions at a ratio of 1 U to 50 μM. Finally, the TG2 was removed by heating over 50 °C and centrifugation.

### Phase transition behavior

The phase transition behavior of LCST-type or UCST-type polypeptides was carried out on a Shimadzu 3600 UV/Vis spectrophotometer (Shimadzu, Japan) equipped with an S-1700 thermoelectric single-cell holder (Shimadzu) in a 1 cm quartz cell. Polypeptide solutions (200 μM unless otherwise mentioned) were equilibrated until no change in transmittance was observed. Transmittance was recorded as a function of temperature at 1 °C min^−1^ and a fixed 600 nm wavelength. The LCST or UCST for each experiment was defined as the temperature at which the transmittance was 50%.

### Cell culture

Cancer cell lines HeLa and MCF-7 were purchased from cell culture center of Institute of Basic Medical Sciences, Chinese Academy of Medical Sciences (Beijing, China). Cell lines HeLa and MCF-7 were cultured in Dulbecco’s modified Eagle’s medium (DMEM, Hyclone, Logan, UT) supplemented with 10% heat-inactivated fetal bovine serum (FBS, Hyclone) and 100 U ml^−1^ penicillin and 100 μg ml^−1^ streptomycin in a humidified incubator in an atmosphere containing 5% CO_2_ (Thermo, Waltham, MA, USA). Cell line SH-SY5Y was cultured for 24 h under normoxic (Basal) or hypoxic (CoCl_2_, 200 μM) conditions in Eagle’s minimal essential medium (Gibco, Grand Island, NY, USA) supplemented with 1% pyruvic acid sodium, 1% nonessential amino acid, 15% FBS (Hyclone), and 100 U ml^−1^ penicillin and 100 μg ml^−1^ streptomycin. The cell lines had been authenticated utilizing short tandem repeat DNA profiling. All cells were tested negative for cross-contamination of other human cells and mycoplasma contamination.

### Immunoblotting

Cells were collected and washed once in PBS. Cells were then lysed in a buffer containing 50 mM Tris-HCl, pH 7.2, 150 mM NaCl, 10 mM MgCl_2_, 1% Triton X-100, 0.5% sodium deoxycholate, 0.1% SDS, 50 mM NaF, 2 mM Na_3_VO_4_ and protease and phosphatase inhibitor cocktail. Protein concentration was determined by Bio-Rad Bradford protein assay and equal amounts of protein were subjected to SDS–PAGE and transferred to nitrocellulose membranes. After blocking with 5% milk or BSA in TBS-T, membranes were incubated with primary antibodies in blocking buffer: mouse anti-β-actin (1:1000; YESEN #30101ES50) and rabbit anti-TGase (1:200; Pierce #PA5-23219) overnight at 4 °C. Proteins were detected with horseradish peroxidase-conjugated secondary antibodies through enhanced chemiluminescence on a Typhoon Trio Variable Mode Imager. Band density was calculated using NIH Image J software. Uncropped gel images are shown in Supplementary Figs. 53 and 54.

### The phase bioseperation of intracellular polypeptides

With the same incubation procedure above, HeLa cells that treated with FTIC labeled peptide 4 were washed by PBS twice and lysed by water. Then, the cell lysates were centrifuged to remove the cell debris, whereas the supernatants were collected for lyophilization. After that, the lyophilized powder was redissolved into 500 μl DI water. The resulting solution was cooled down to 4 °C and the precipitate was collected by removing the supernatants. Recycling the above procedures for four times, high-quality polypeptide was obtained.

### The intracellular polymerization and self-aggregation process

The FTIC labeled peptide 4 with a concentration of 600 μM was subjected to HeLa cells (20,000 cells per well). At time-scale of 0.5–24 h, the FITC-labeled polypeptides were obtained according to the thermoprecipitation method. The MWs of the obtained polypeptides were measured by GPC. Temperature-dependent and time-dependent experiments were conducted on a confocal laser scanning microscope (Zeiss LSM710) to monitor the polymerization and self-aggregation process. The cooling speed for **P4** solution was 1 °C per minute. The time-dependent monitoring of **P7** and **P9** were obtained at 37 °C.

### Quantification of the retention efficiency

The standard curve of FITC-labeled peptide monomers was carried out by UV–vis spectra at a wavelength at 491 nm. After inducing FITC-labeled peptide monomers to HeLa and MCF-7 cells (8000 cells per well) with an original concentration of 600 μM at a time range of 0.5–24 h, the entrapped peptides inside cells was quantified through cell lysates. The retention efficiency of peptides was calculated with the molecular percentage of peptides blocked inside cells to the feeding peptides.

### In vivo therapeutic experiments

Animal experiments were carried out complying with NIH guidelines for the care and euthanasia of laboratory animals of National Center for Nanoscience and Technology Animal Study Committee’s requirement and according to the protocol approved by the Institutional Animal Care. The mice were random allocation to each group. To establish tumors in 6-week-old female BLAB/c nude mice, two million HeLa cells suspended in 50 ml PBS (pH 7.4) were injected subcutaneously in the hinder leg of the mouse. Tumors were grown until a volume of 50–100 mm^3^, and then treatment that consisted of 5.0 mg kg^−1^ control peptide, peptide 9, peptide 10, or saline was initiated through intravenous administration, every other day for 2 weeks. Total mice body weights and tumor sizes (length and width by caliper) were measured every other day. Tumor volumes were calculated assuming an ellipsoid shape with the formula (length × width^2^)/2. At day 14, the mice were killed and the tumors were collected, fixed and stained for bioTEM analysis. The statistical significance between all pairs of components was assessed using one-way analysis of variance followed by post hoc Tukey’s test.

### Data availability

The data that support the findings of this study are available within the article, its Supplementary Information files and from the corresponding author upon reasonable request. Results presented here can be found at: https://figshare.com/s/74869ef752f2490ecc1f.

## Electronic supplementary material

Supplementary Information
