# Supplementary material for: Intracellular construction of topology-controlled polypeptide nanostructures with diverse biological functions
Source: Nat Commun. 2017 Nov 2;8:1276. doi: 10.1038/s41467-017-01296-8 (PMC5668255; doi:10.1038/s41467-017-01296-8)
Supplement: Supplementary file 1 — Supplementary Information [file 41467_2017_1296_MOESM1_ESM.pdf]

Supplementary Figures

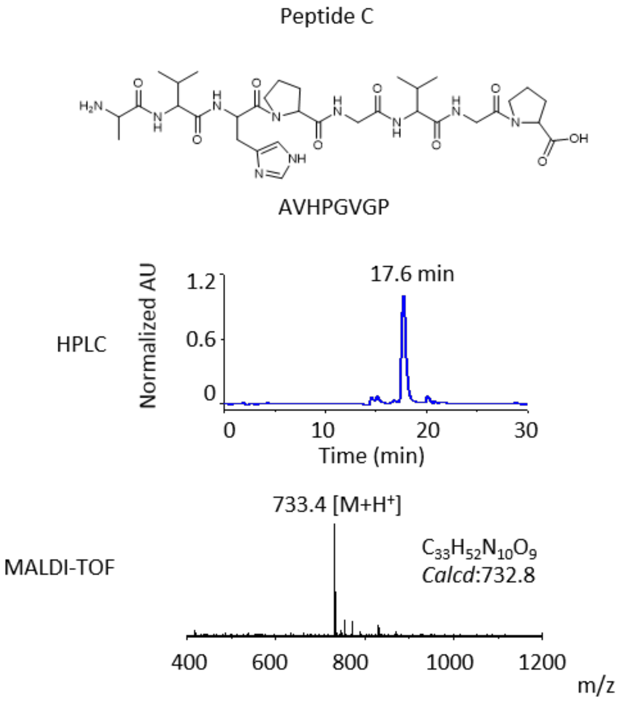

Supplementary Figure 1. Characterizations of peptide control by HPLC and MALDI-TOF.

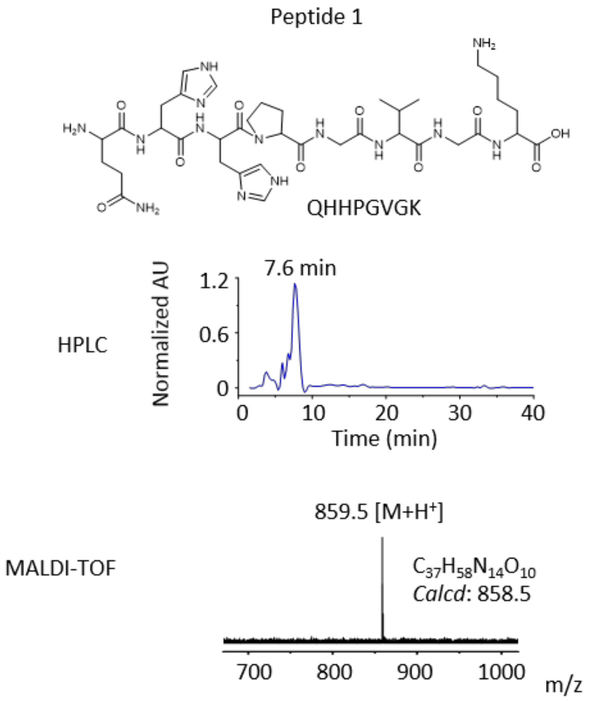

Supplementary Figure 2. Characterizations of peptide 1 by HPLC and MALDI-TOF.

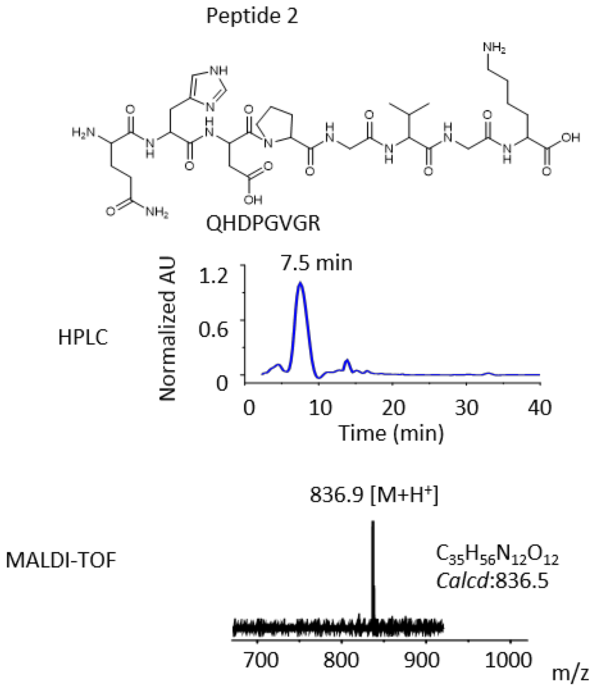

Supplementary Figure 3. Characterizations of peptide 2 by HPLC and MALDI-TOF.

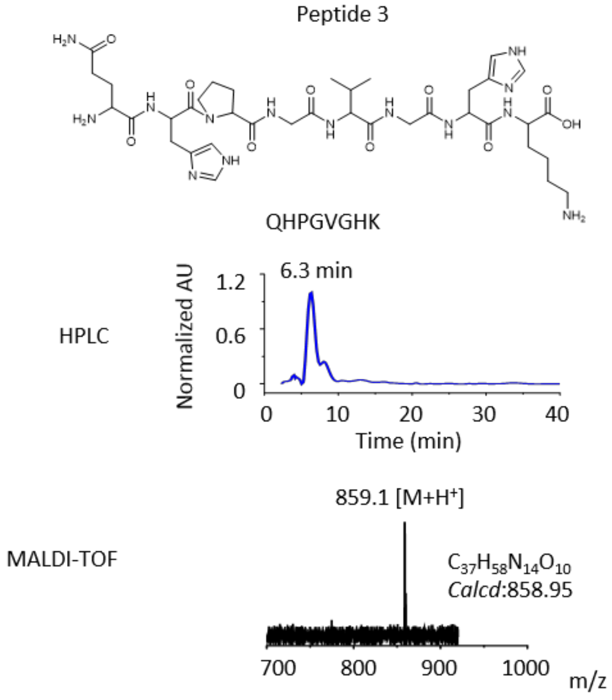

Supplementary Figure 4. Characterizations of peptide 3 by HPLC and MALDI-TOF.

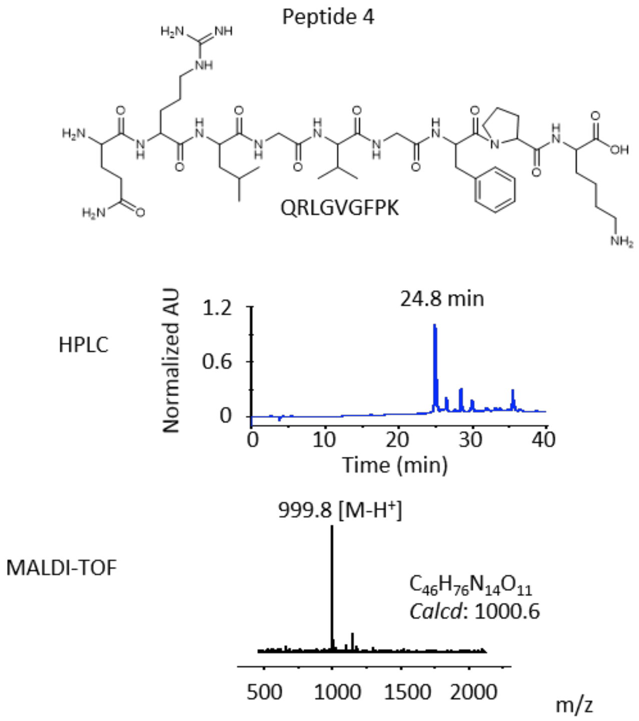

Supplementary Figure 5. Characterizations of peptide 4 by HPLC and MALDI-TOF.

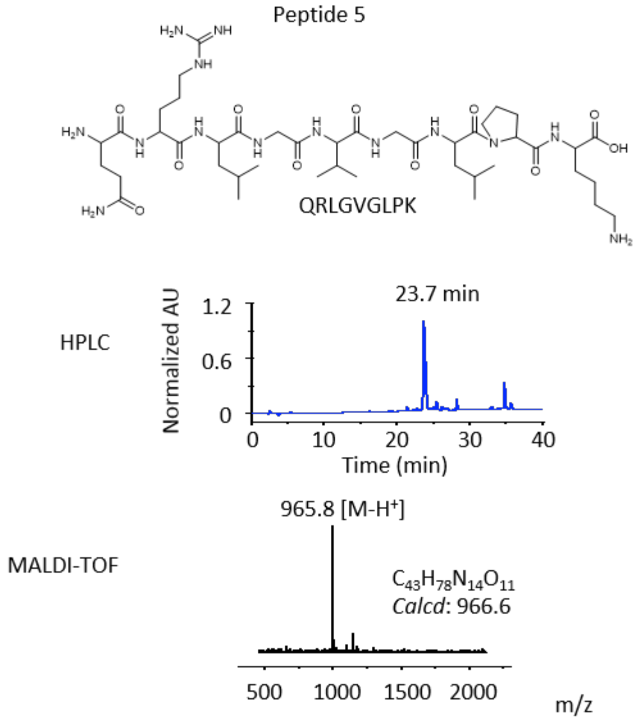

Supplementary Figure 6. Characterizations of peptide 5 by HPLC and MALDI-TOF.

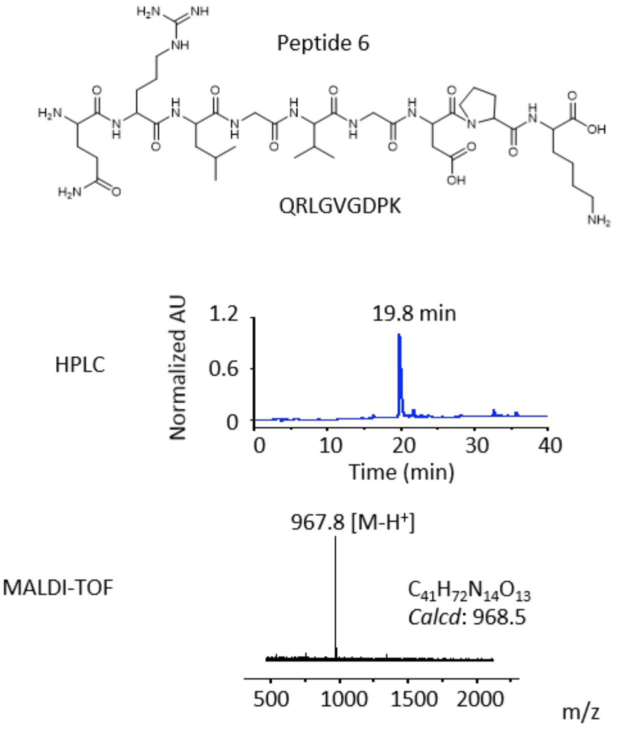

Supplementary Figure 7. Characterizations of peptide 6 by HPLC and MALDI-TOF.

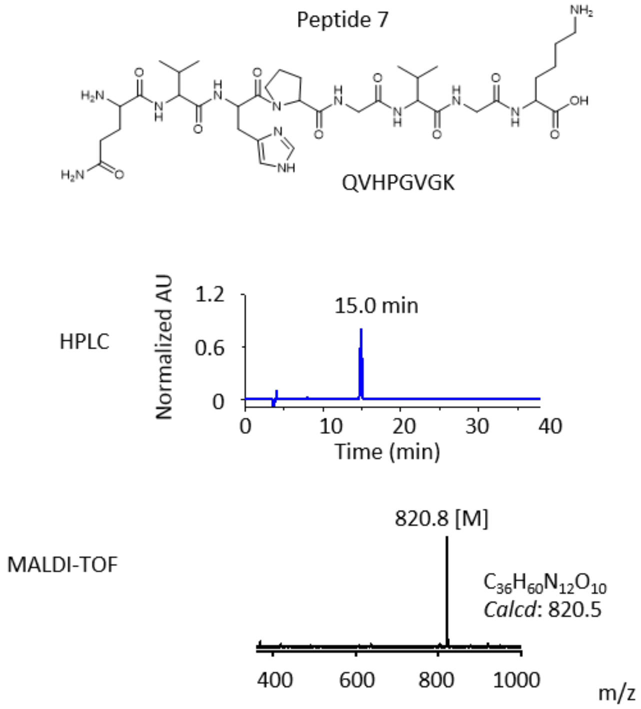

Supplementary Figure 8. Characterizations of peptide 7 by HPLC and MALDI-TOF.

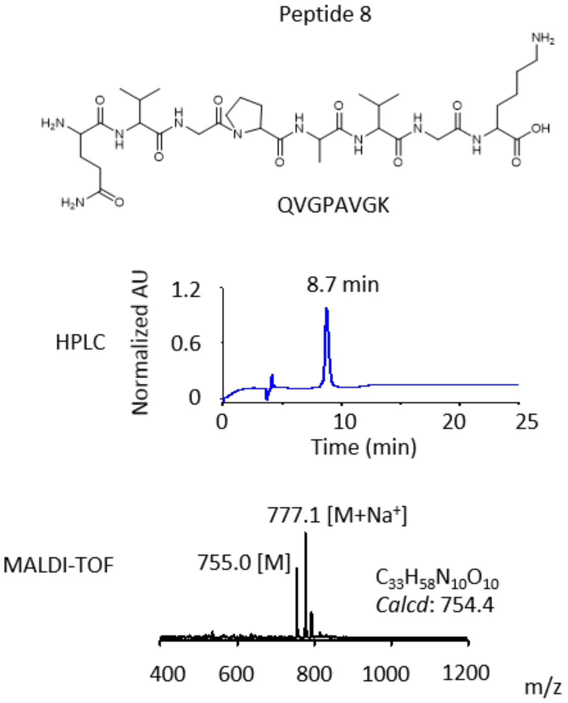

Supplementary Figure 9. Characterizations of peptide 8 by HPLC and MALDI-TOF.

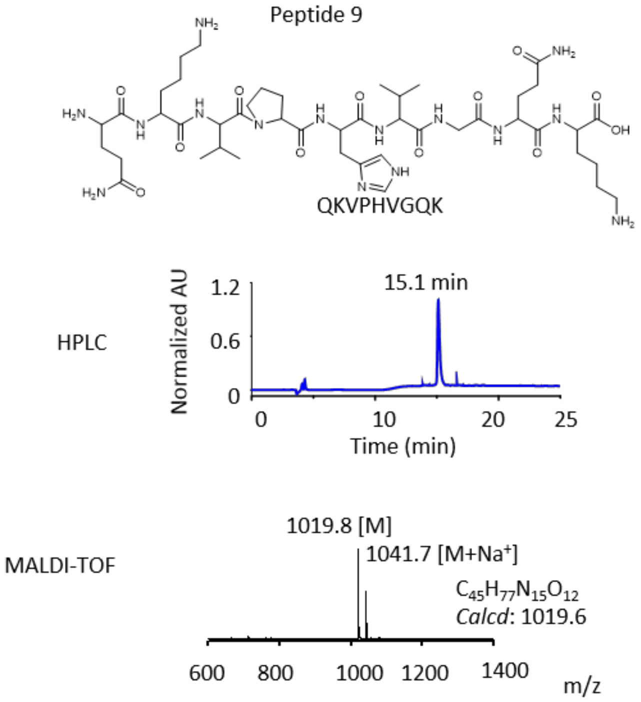

Supplementary Figure 10. Characterizations of peptide 9 by HPLC and MALDI-TOF.

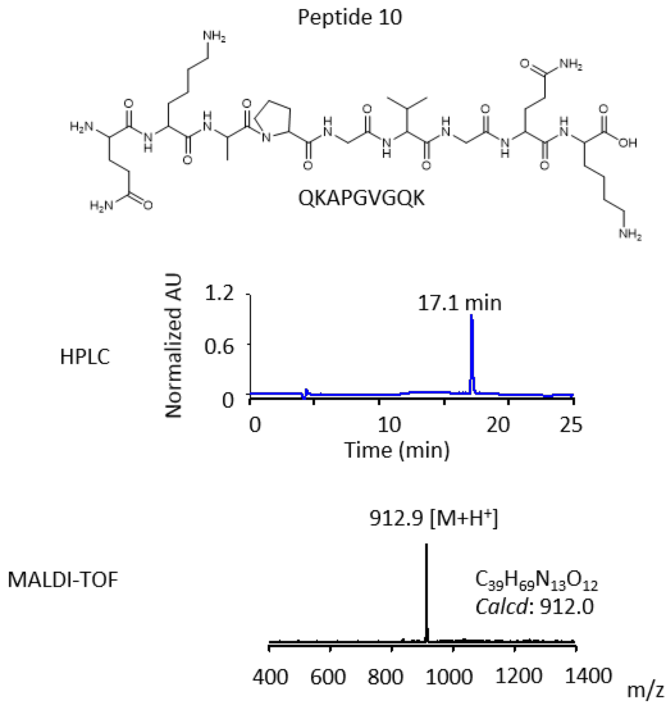

Supplementary Figure 11. Characterizations of peptide 10 by HPLC and MALDI-TOF.

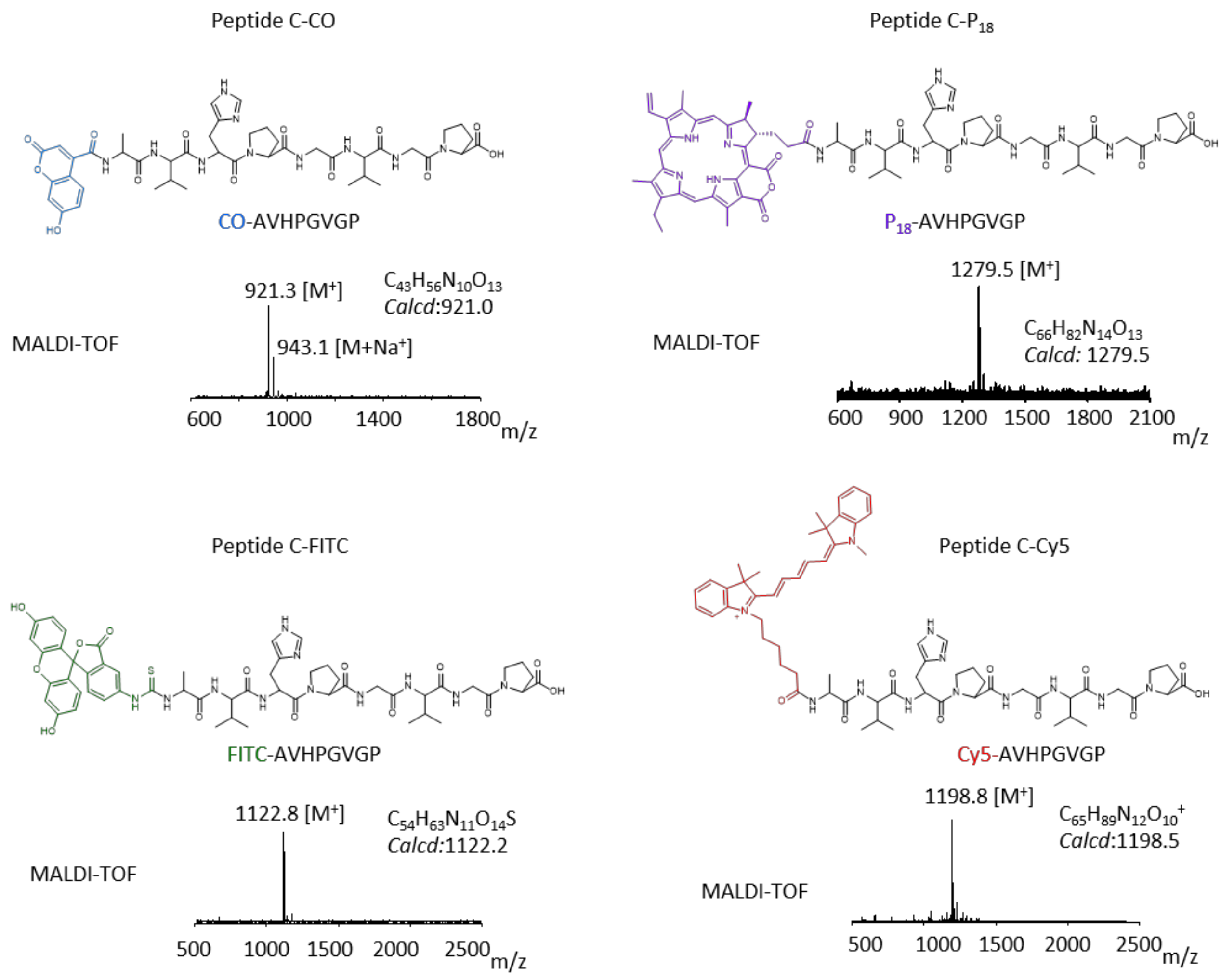

Supplementary Figure 12. Characterizations of peptide C labeled with CO, P_{18}, FITC and Cy5 by MALDI-TOF.

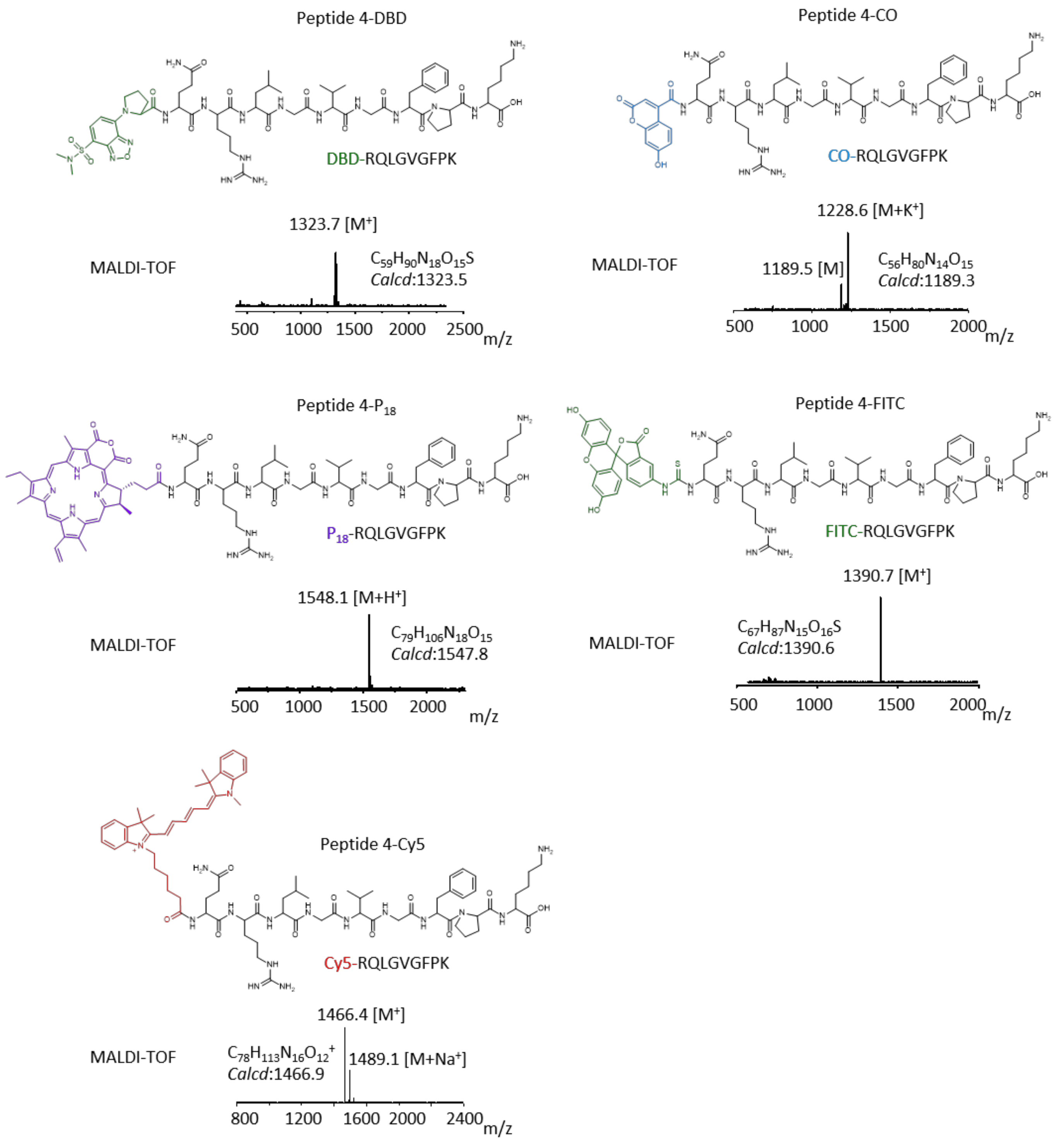

Supplementary Figure 13. Characterizations of peptide 4 labeled with DBD, CO, P_{18}, FITC and Cy5 by MALDI-TOF.

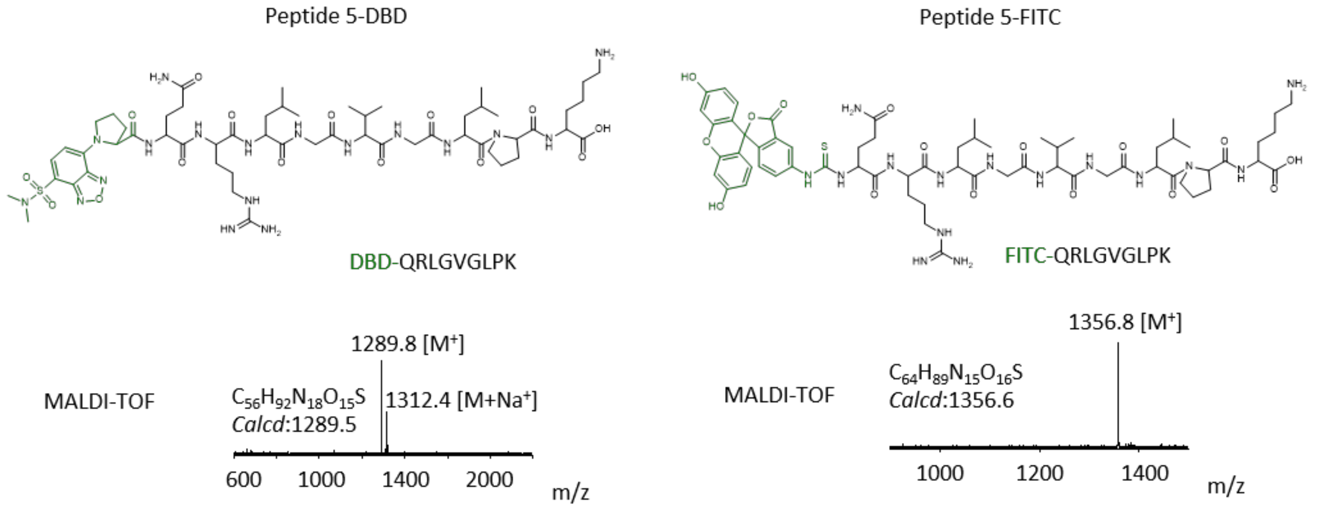

Supplementary Figure 14. Characterizations of peptide 5 labeled with DBD and FITC by MALDI-TOF.

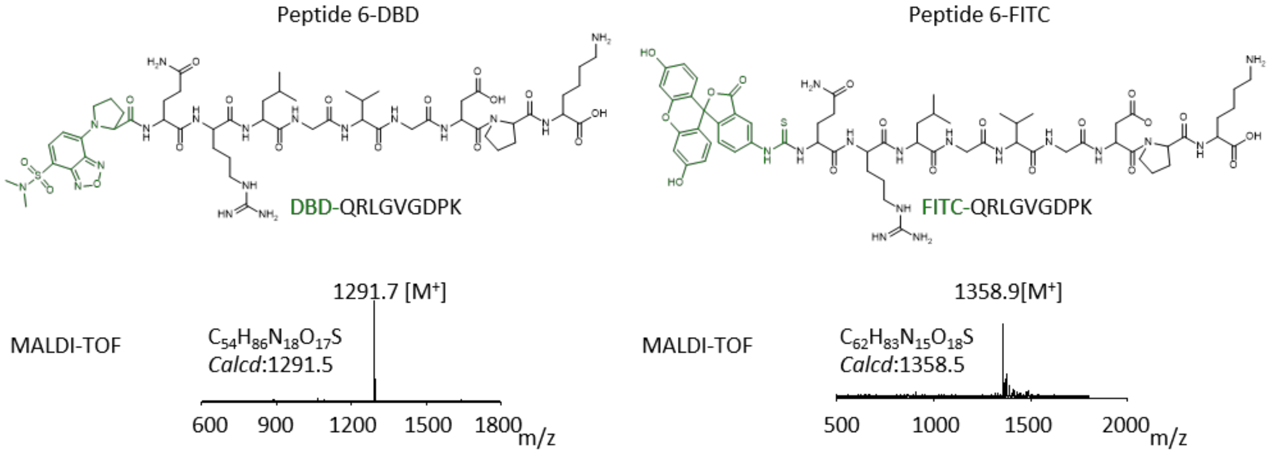

Supplementary Figure 15. Characterizations of peptide 6 labeled with DBD and FITC by MALDI-TOF.

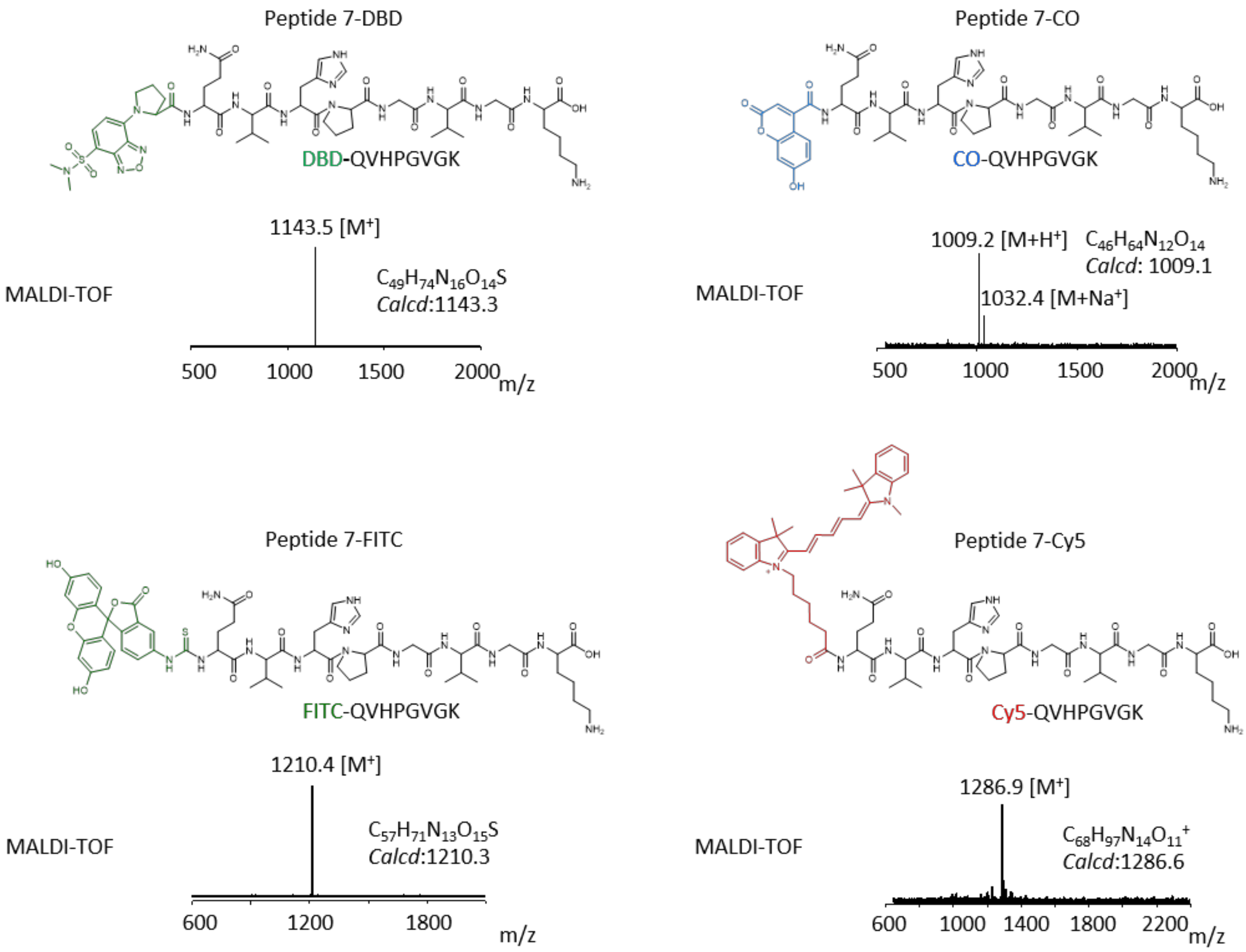

Supplementary Figure 16. Characterizations of peptide 7 labeled with DBD, CO, FITC and Cy5 by MALDI-TOF.

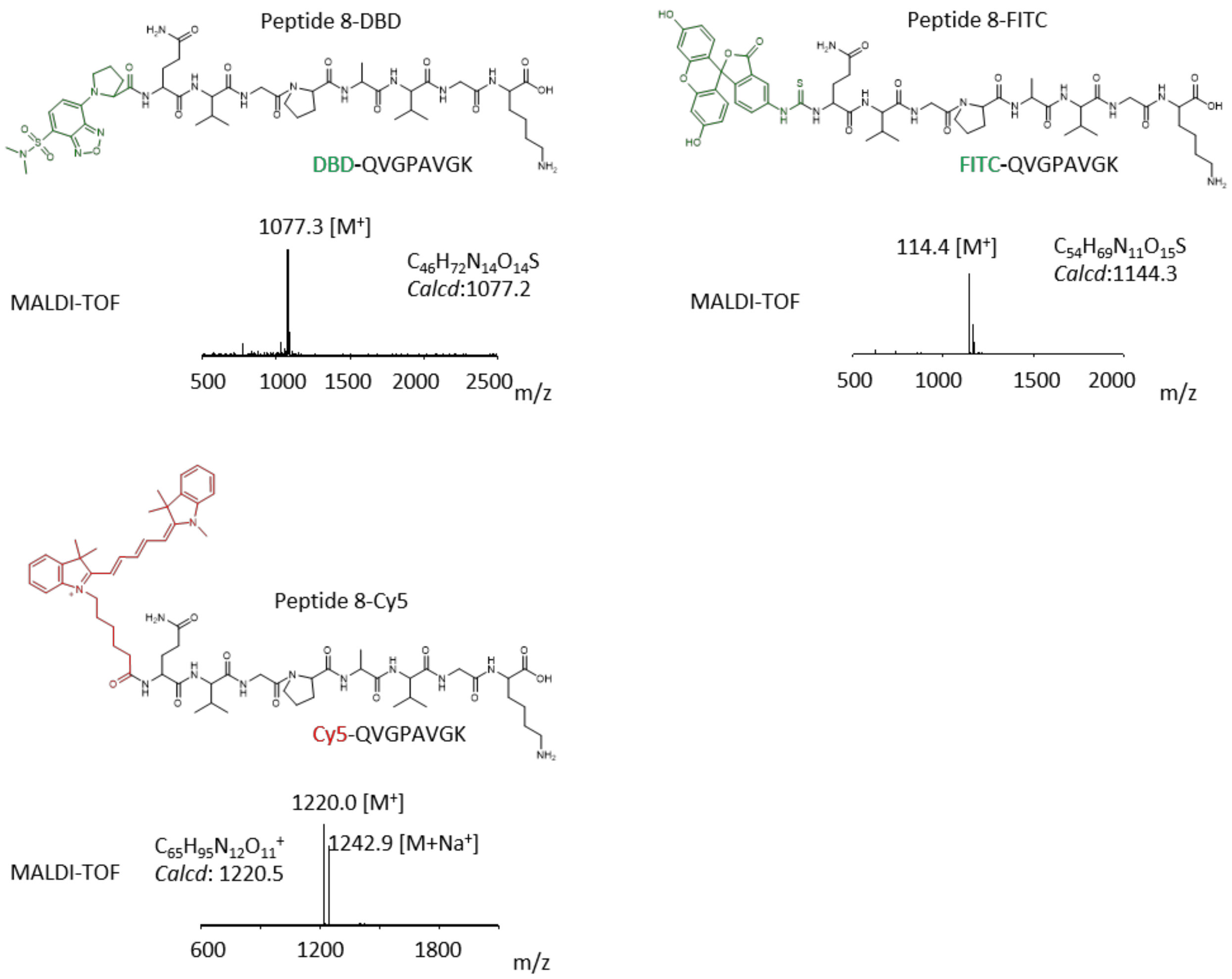

Supplementary Figure 17. Characterizations of peptide 8 labeled with DBD, FITC and Cy5 by MALDI-TOF.

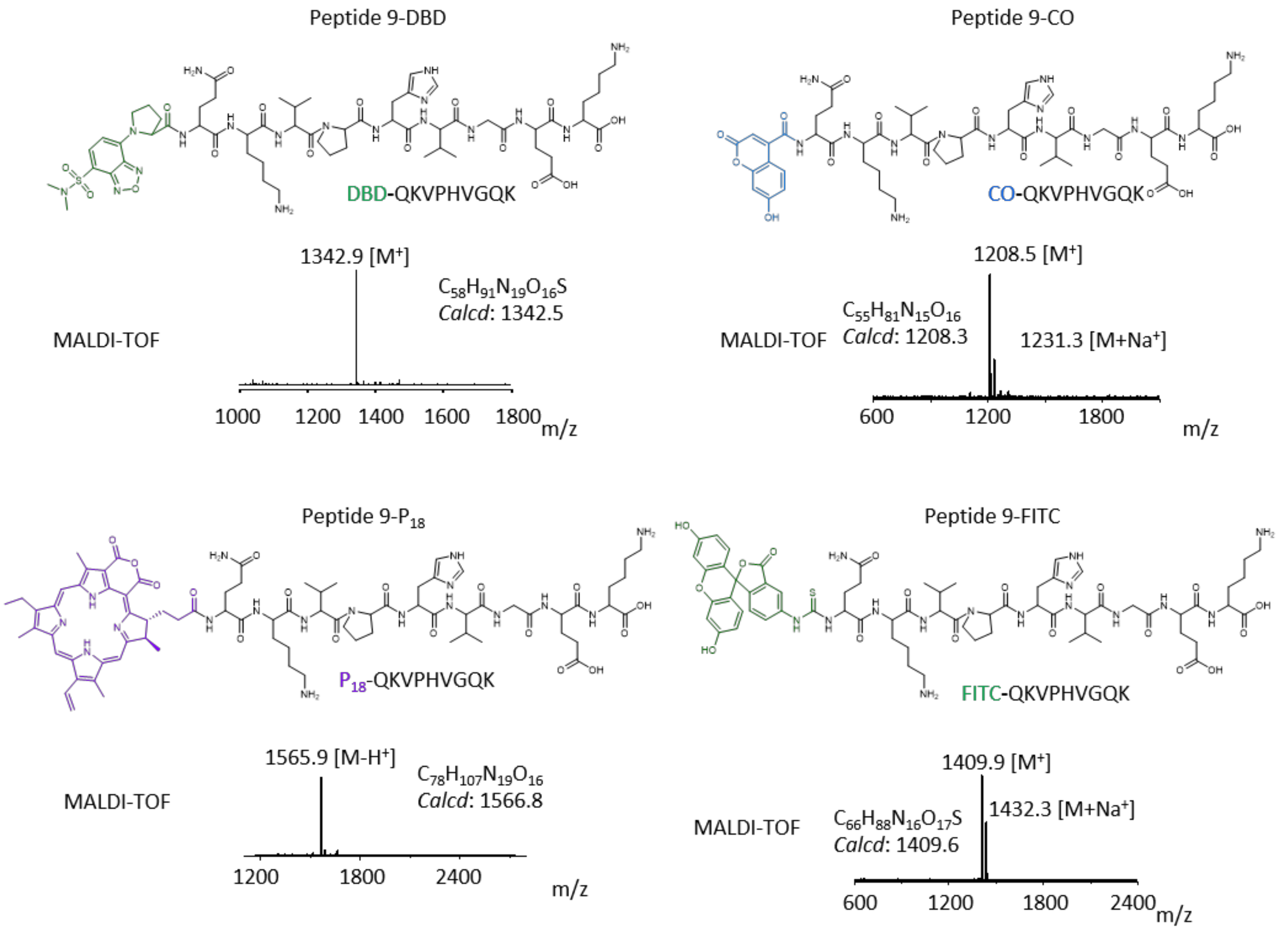

Supplementary Figure 18. Characterizations of peptide 9 labeled with DBD, CO, P_{18} and FITC by MALDI-TOF.

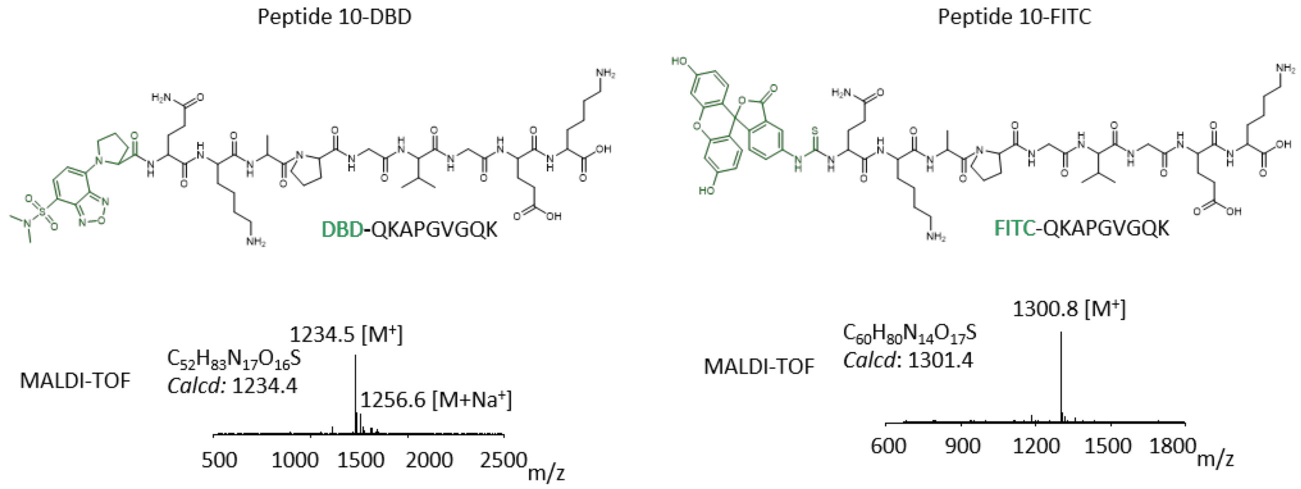

Supplementary Figure 19. Characterizations of peptide 10 labeled with DBD and FITC by MALDI-TOF.

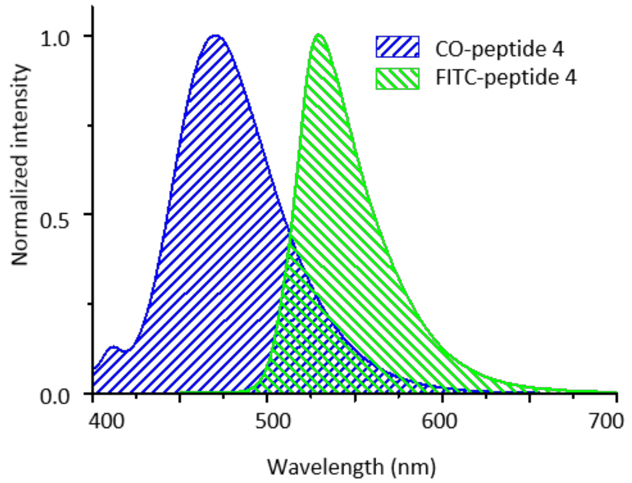

Supplementary Figure 20. Fluorescence spectra of FITC-peptide 4 and Coumarin-peptide 4.

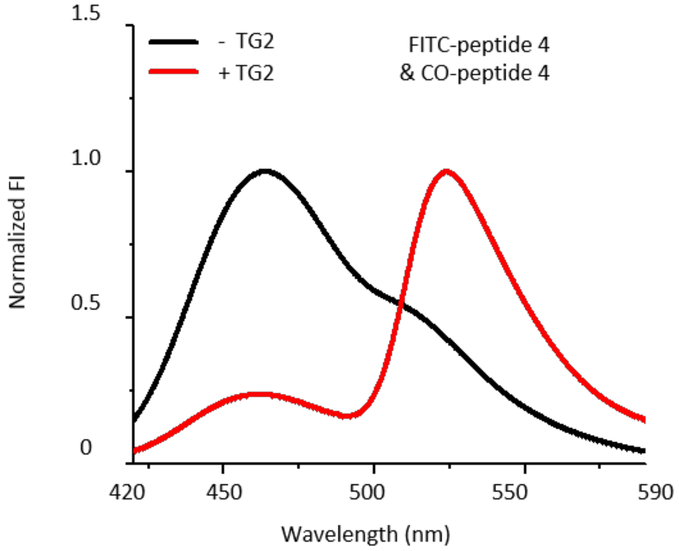

Supplementary Figure 21. The fluorescence spectra of the mixture of FITC-peptide 4 and CO-peptide 4 with absence/presence of TG2 in buffer at 37 ^{0}C for 12 h.

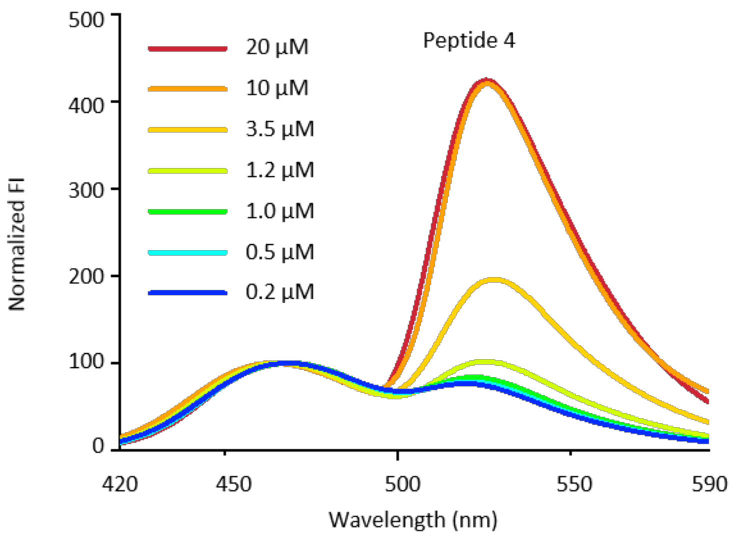

Supplementary Figure 22. The fluorescence spectra of different concentration of peptide substrate (molecule ratio of FITC-peptide 4 to CO-peptide 4 was 1:1) treated with 1 U TG2.

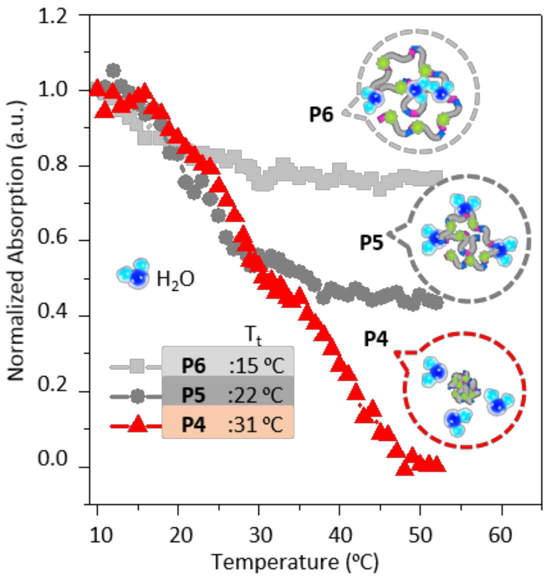

Supplementary Figure 23. The temperature-dependent turbidimetry for polypeptides. The insert table listed the transition temperatures (Tt) for **P 4**-**P6**.

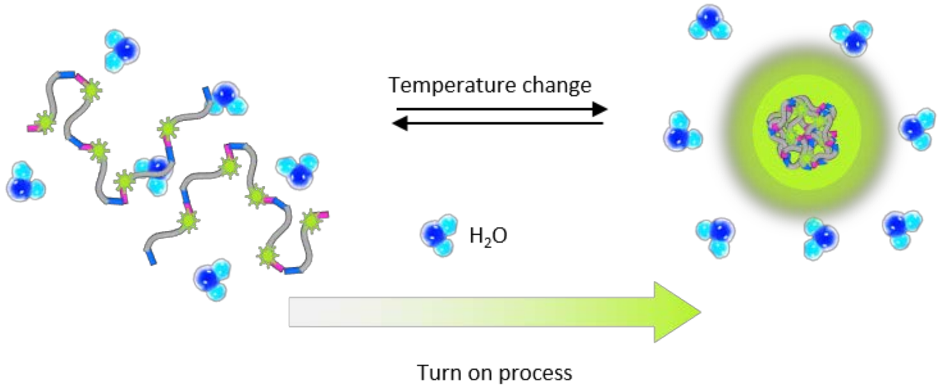

Supplementary Figure 24. Functional diagram of fluorescence nanoaggregates formation in aqueous medium.

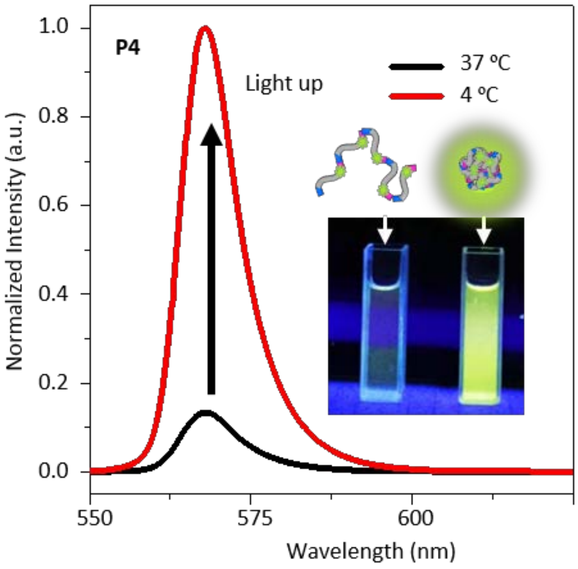

Supplementary Figure 25. The thermosensitive light up of **P4** (200 μM) from 37 °C to 4 °C. The pictures of fluorescence images of **P4** before/after collapsed were inserted.

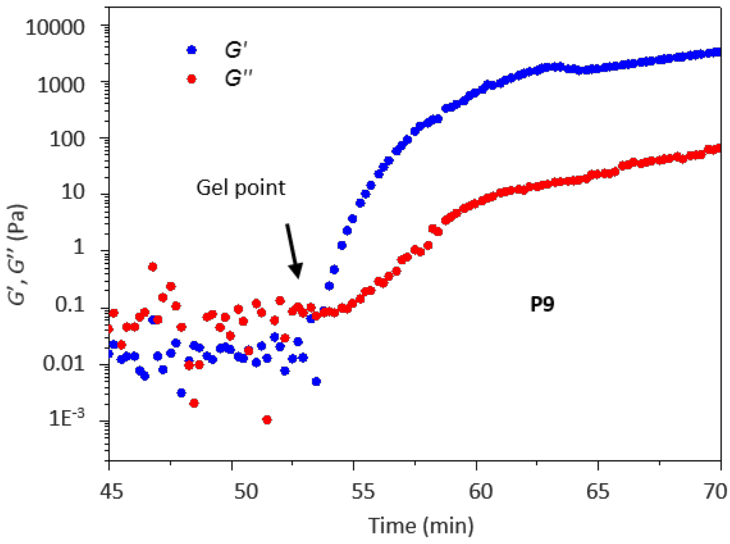

Supplementary Figure 26. Dynamic time sweep rheological analysis of **P9** at a fixed frequency (0.5 Hz) and strain (5 %).

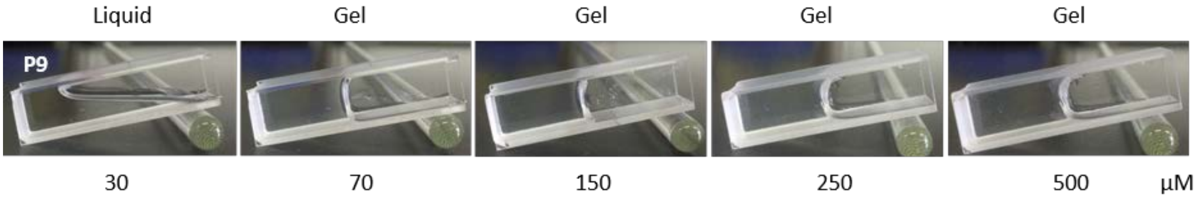

Supplementary Figure 27. Determination of minimum gelation concentration of **P9** by inverted vial method under 20 °C.

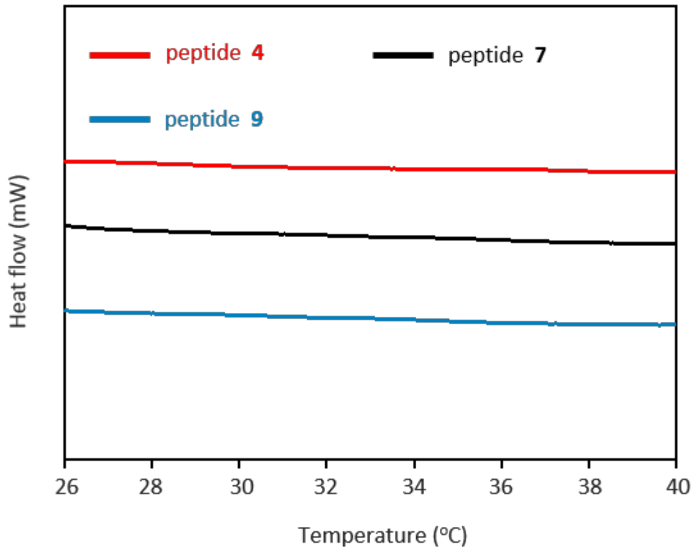

Supplementary Figure 28. Differential scanning calorimetry (DSC) thermogram of peptide 4, 7 and 9 at a temperature ramp rate of 2 °C min^{-1}.

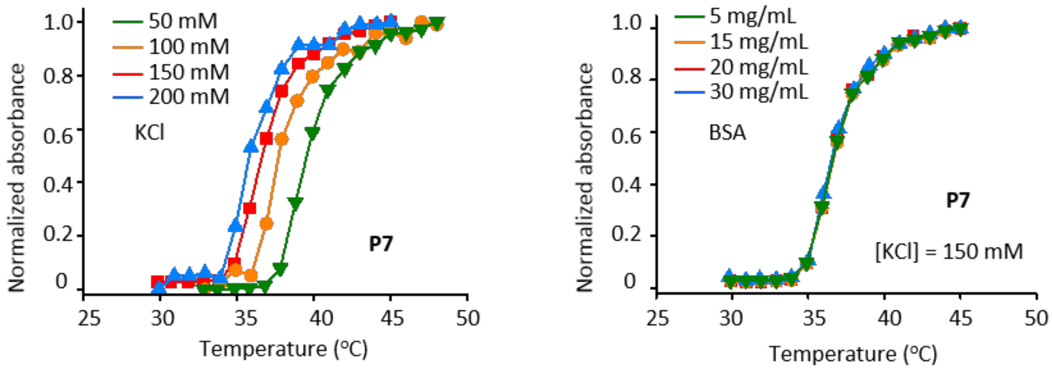

Supplementary Figure 29. The thermosensitivity and phase transition property of **P7** in KCl solutions with concentrations of 50, 100, 150 and 200 mM and BSA (contained150 mM KCl) solutions with concentrations of 5, 15, 20 and 30 mg ml^{-1} .

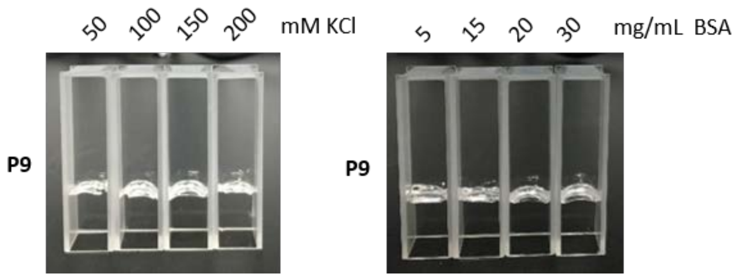

Supplementary Figure 30. The thermosensitivity and phase transition property of **P9** at 37 °C in KCl solutions with concentrations of 50, 100, 150 and 200 mM and BSA (contained150 mM KCl) solutions with concentrations of 5, 15, 20 and 30 mg ml^{-1} .

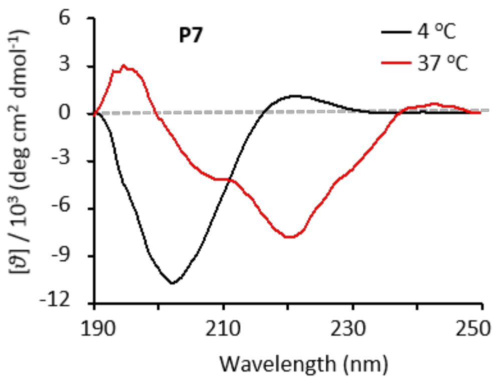

Supplementary Figure 31. CD spectra of TG2 and **P7** at 4 °C and 37 °C with a concentration of 0.5 µM.

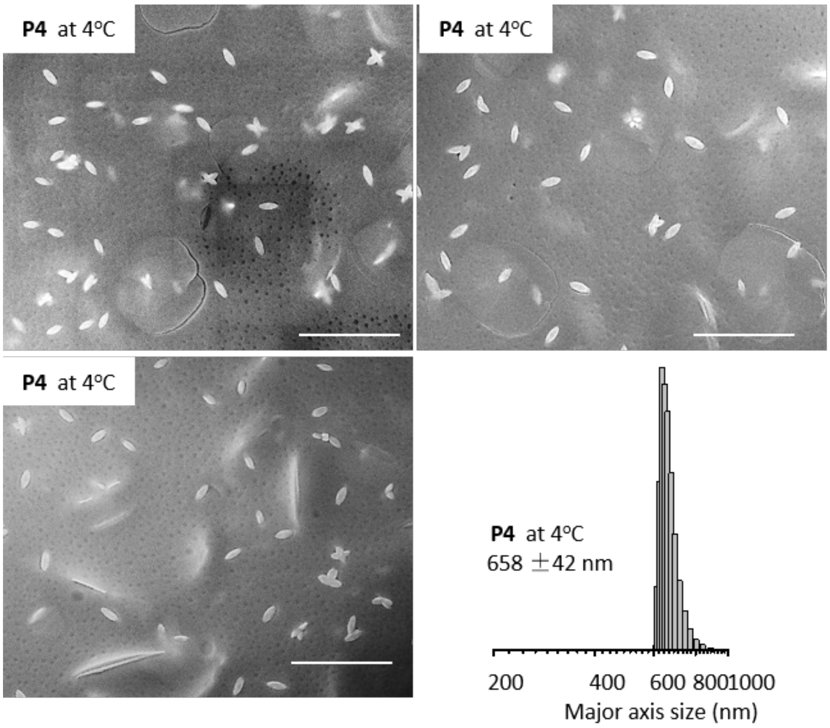

Supplementary Figure 32. Morphology and size distribution (the statistic was obtained from SEM images) of nanoaggregates of **P4** at 4 ^{o}C. *Scale bar*, 4 μm.

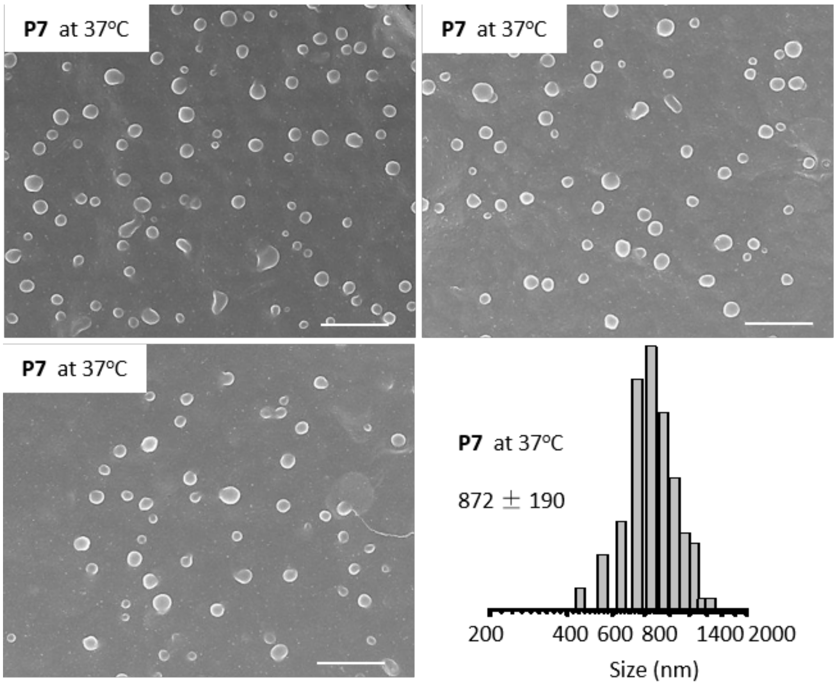

Supplementary Figure 33. Morphology and size distribution (the statistic was obtained from SEM images) of nanoaggregates of **P7** at 37 ^{o}C. *Scale bar*, 5 μm.

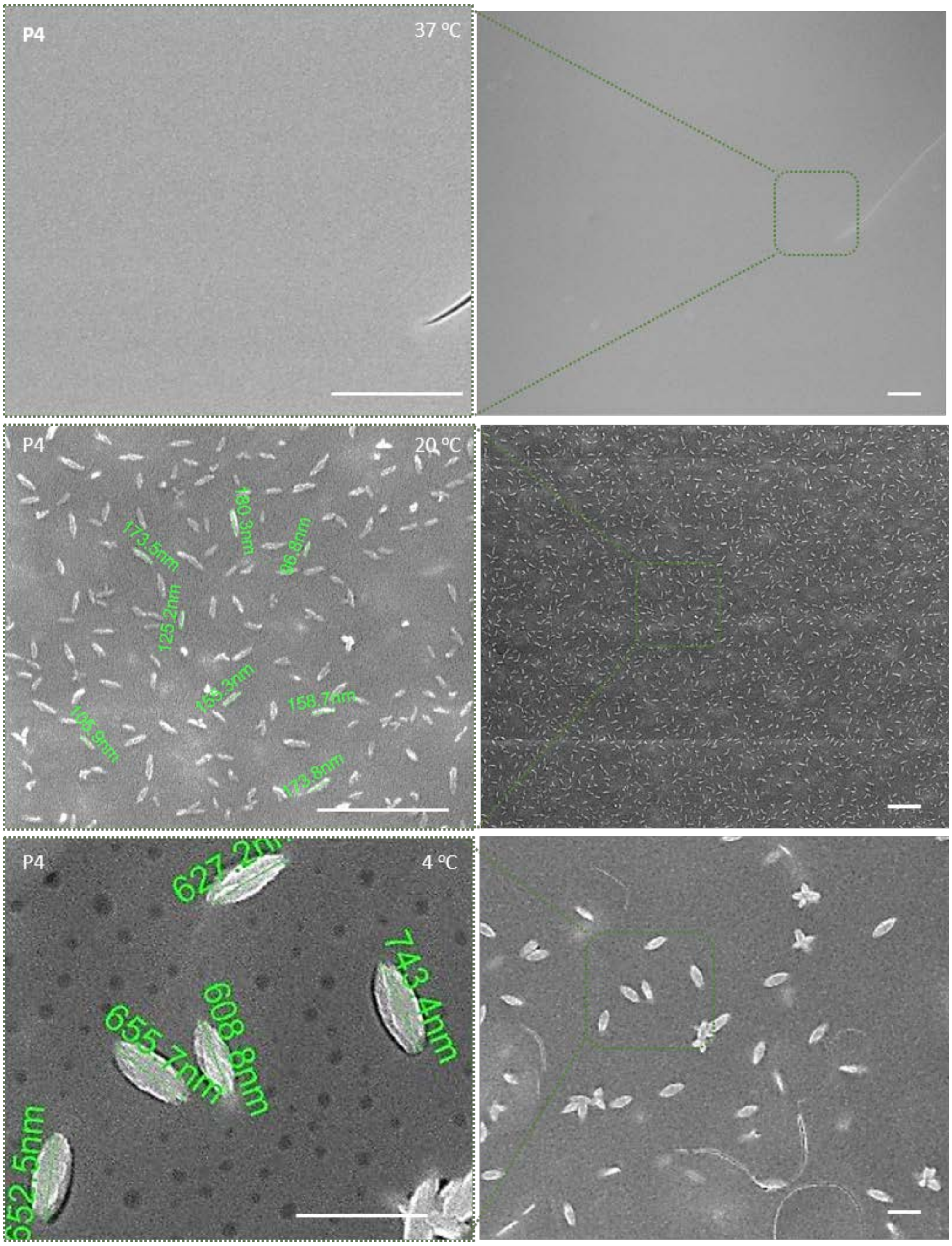

Supplementary Figure 34. The SEM images of self-aggregation process of **P4** during cooling procedure. *Scale bar*, 1 µm.

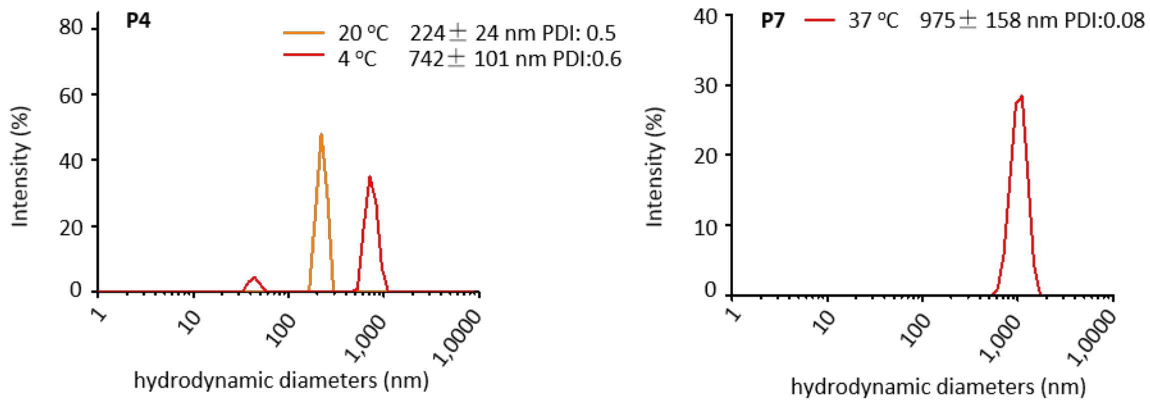

Supplementary Figure 35. The dynamic light scattering (DLS) of the hydrodynamic diameters of the nanoparticles formed by **P4** (at 20 °C and 4 °C) and **P7** (at 37 °C)

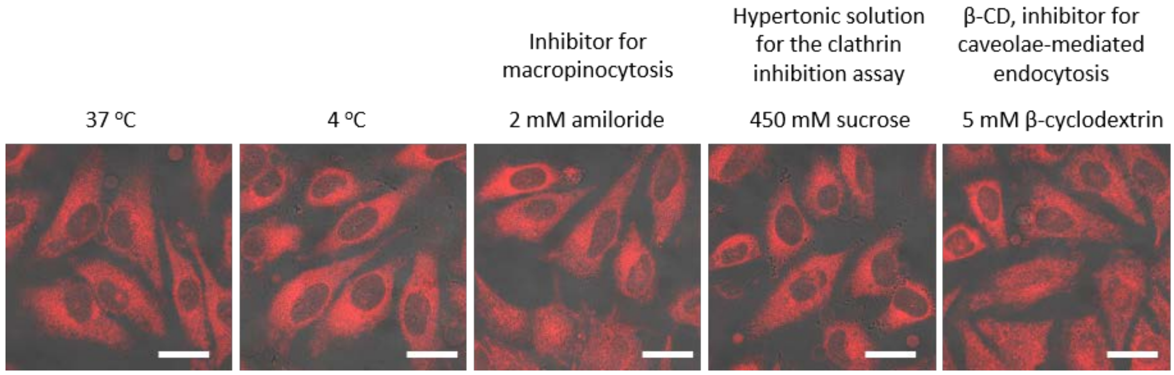

Supplementary Figure 36. The confocal images of HeLa cells treated with peptide control under various conditions in order to identify the internalization pathway. The cells, prior to the addition of the peptides were incubated with PBS under 37 °C and 4 °C, 2 mM amiloride, 450 mM sucrose and 5 mM β-cyclodextrin. *Scale bar*, 30 μm.

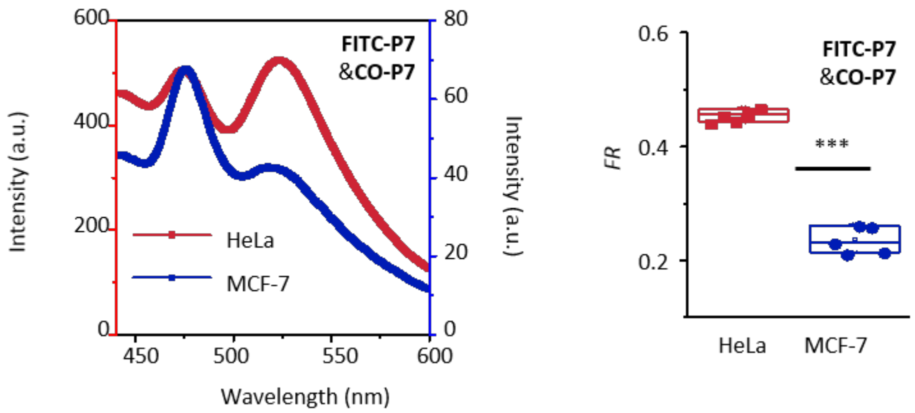

Supplementary Figure 37. Fluorescence spectra and FRET responses (FR) upon introducing mixture of **FITC-P7** and **CO-P7** (molecular ratio of 1:1) to HeLa and MCF-7 cells. The spectra were obtained by the cell lysate. The statistical significances were determined using a two-sample student's *t*-test (*** $p < 0.001$).

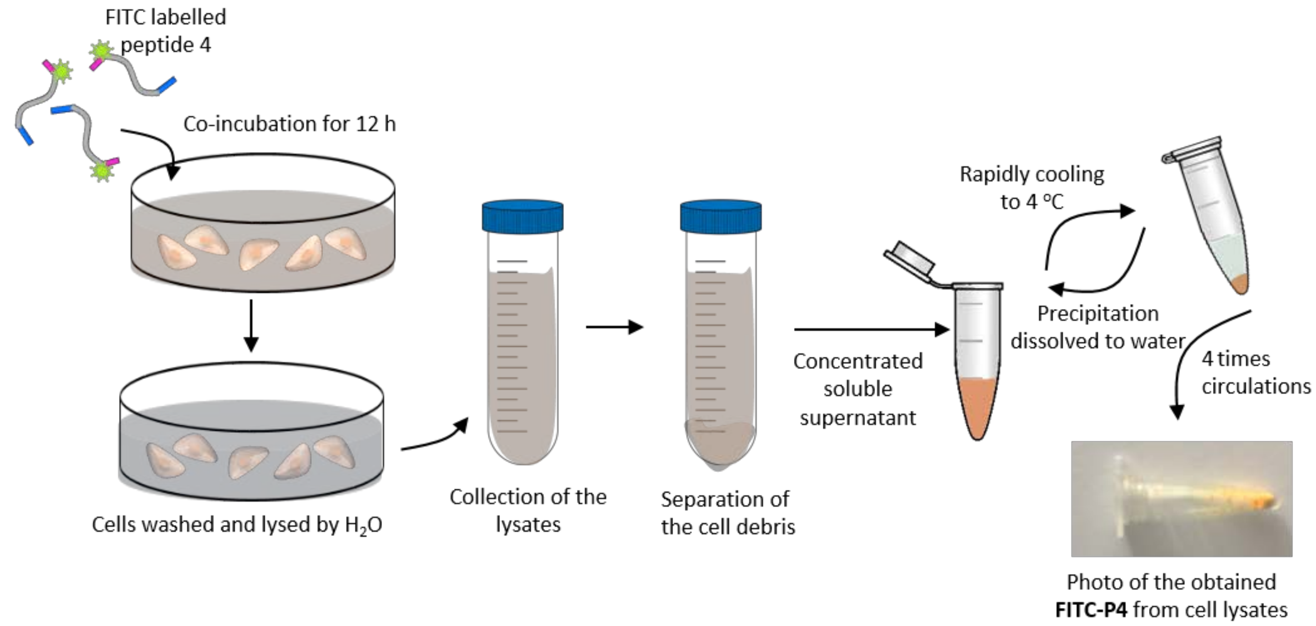

Supplementary Figure 38. The illustration of the phase bioseparation process from cell lysates and the photograph of **FITC-P4** obtained from HeLa cell lysate for the molecule weight measurement by GPC.

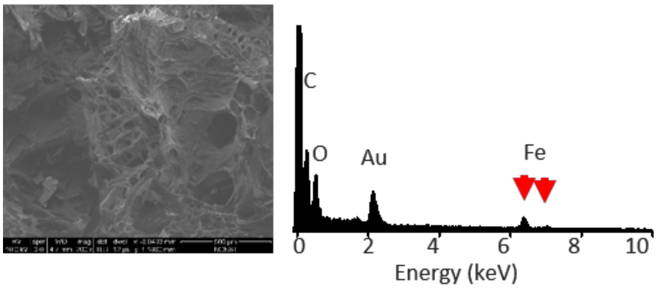

Supplementary Figure 39. The SEM images of the hydrogel obtained from HeLa cell lysate and confirmed by EDS with Fe^{2+} coordinated P_{18} labelling (**P_{18}-P9**).

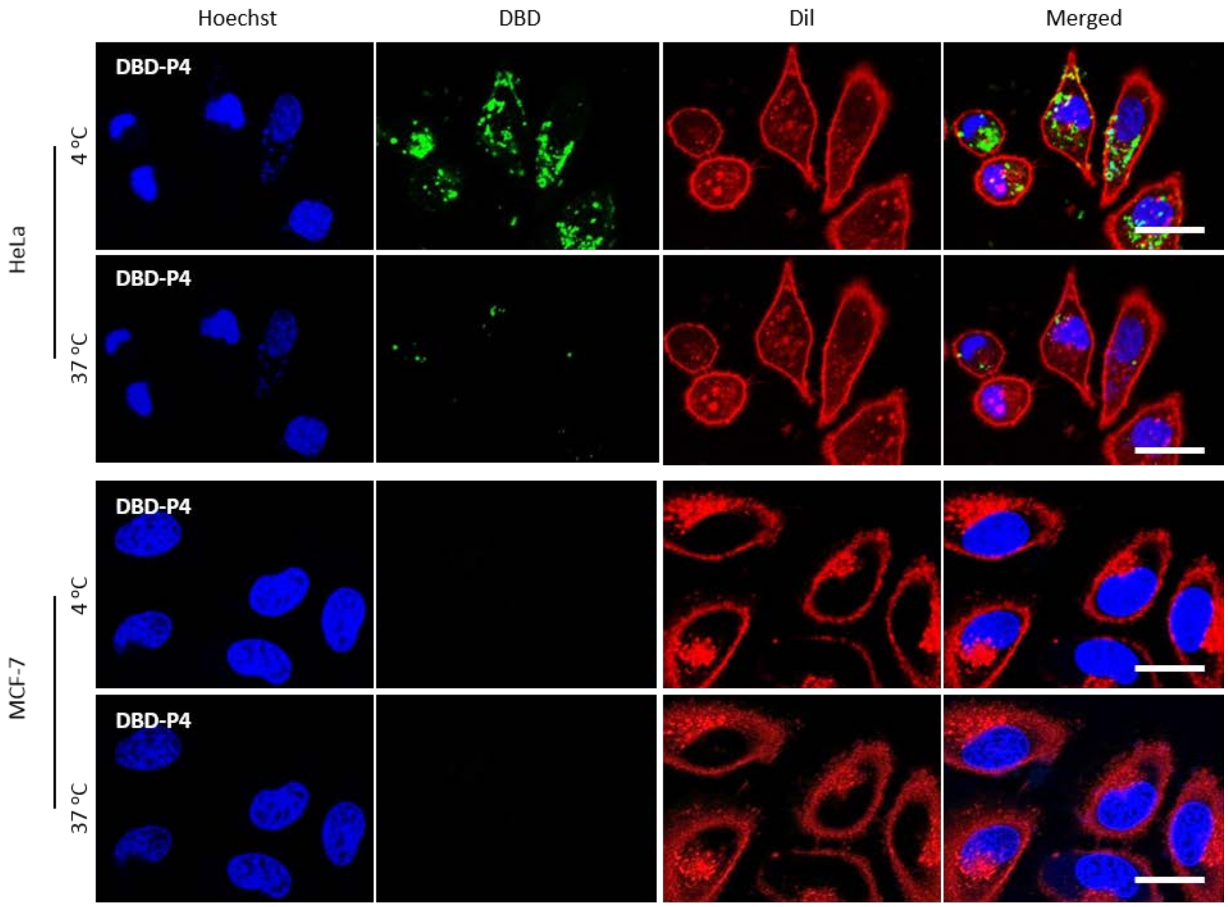

Supplementary Figure 40. The confocal images of TG2 positive cell line (HeLa cells) and negative cell line (MCF-7) co-incubating with **DBD-P4** (600 μM) for 12 h and images were obtained at 4°C and 37°C respectively. The cell nucleus and membrane were stained by Hoechst (blue) and Dil (red), respectively. The green dot indicated for fluorescence nano-aggregates. *Scale bar*, 30 μm.

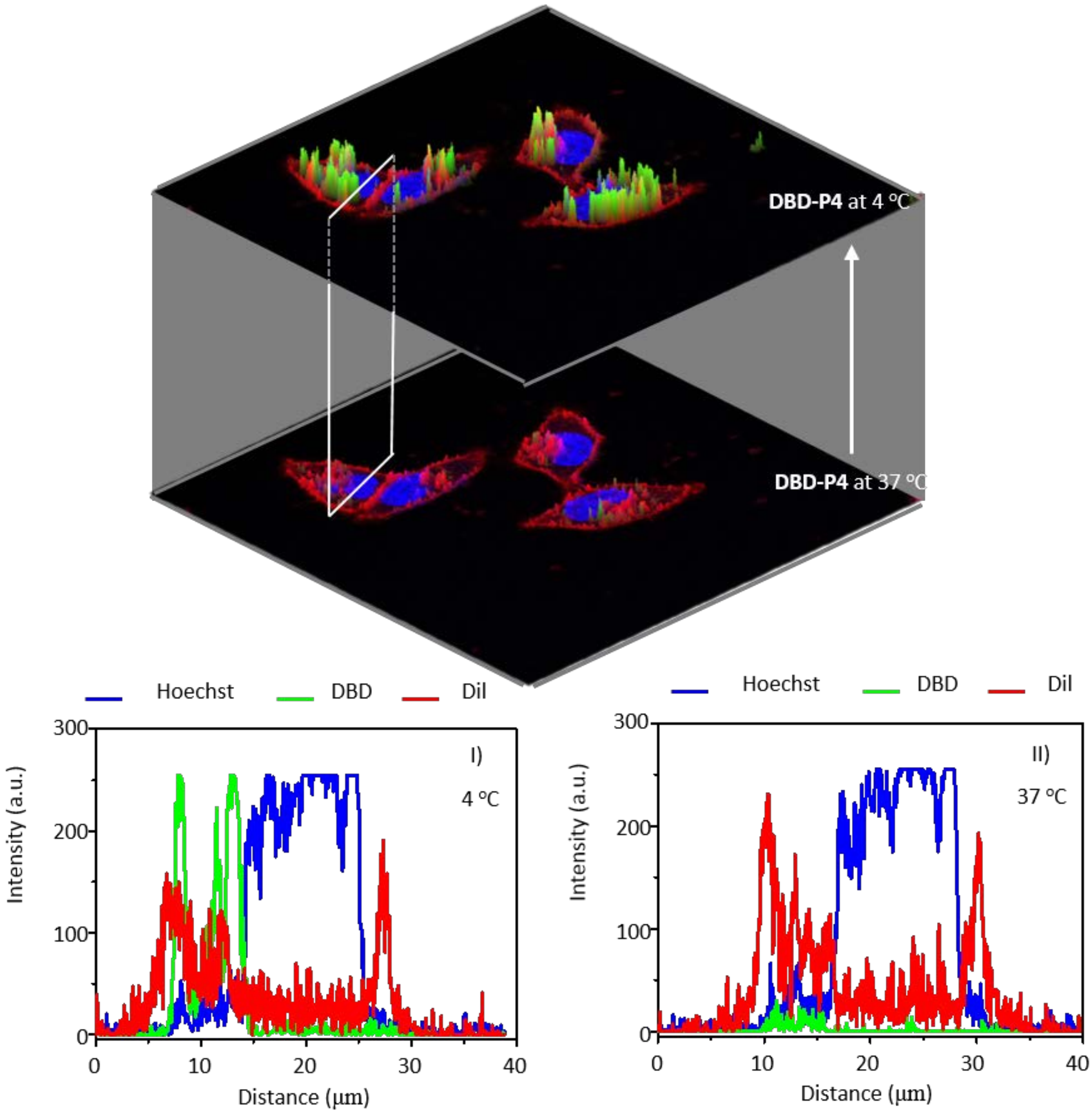

Supplementary Figure 41. The *in situ* polymerization and fluorescence generation upon nano-aggregates formation in cells. Confocal images (3D) of intracellular polymerized **DBD-P4** (green) in extended state (37 °C) and collapsed state (4 °C). The cell nucleus and membranes were labeled by Hoechst (blue) and Dil (red), respectively. The fluorescence intensity profiling of the white lines at 4 °C (I) and 37 °C (II) corresponding to the confocal images.

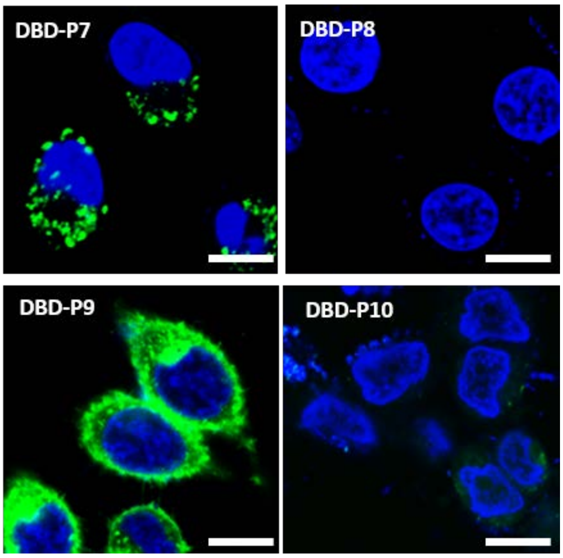

Supplementary Figure 42. Visualization *in situ* isothermal collapse of the **P7**-**P10** inside HeLa cells using DBD labeling. *Scale bar*, 15 μm.

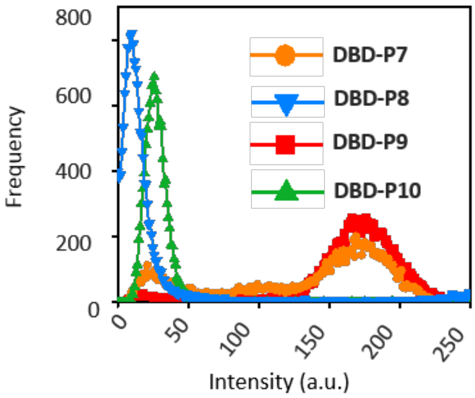

Supplementary Figure 43. The statistical analysis of the fluorescence intensity inside cells after treated with DBD labelled **P7**-**P10**.

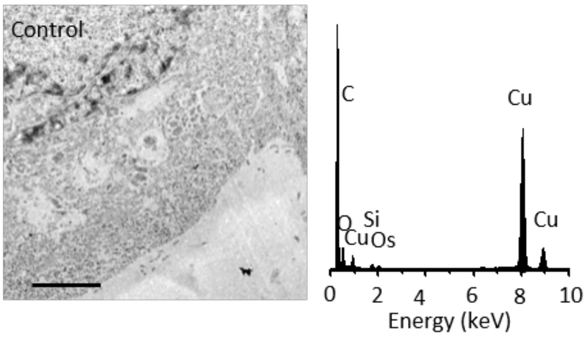

Supplementary Figure 44. TEM image of HeLa control cells without treatment and the EDS in cytoplasm. *Scale bar*, 1 μm.

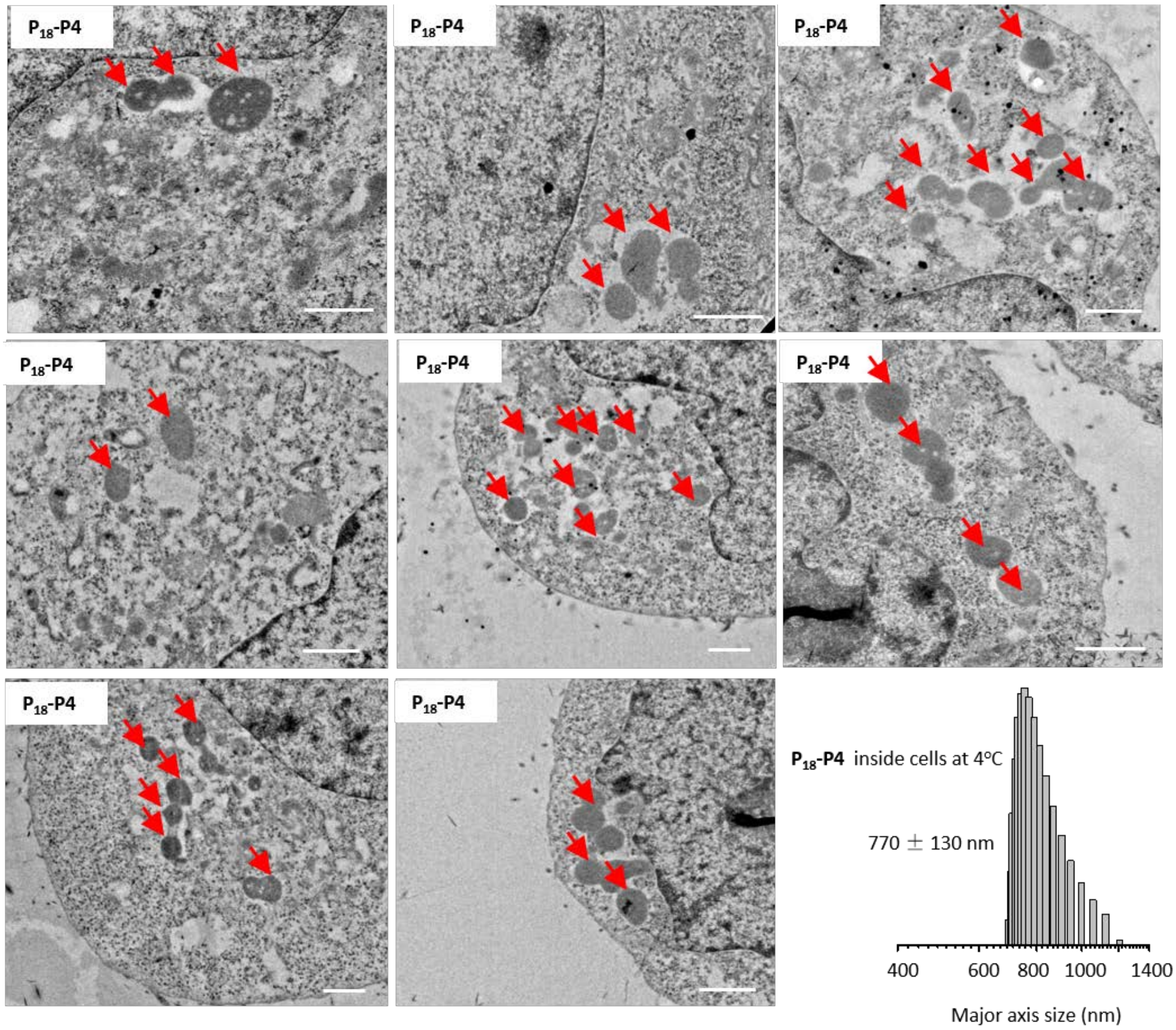

Supplementary Figure 45. The TEM images of HeLa cell ultrathin sections after treated with P_{18} labelled peptide 4 and the statistical size distribution of the nanoaggregates (red arrows) calculated on TEM images at 4°C. *Scale bar*, 1 µm.

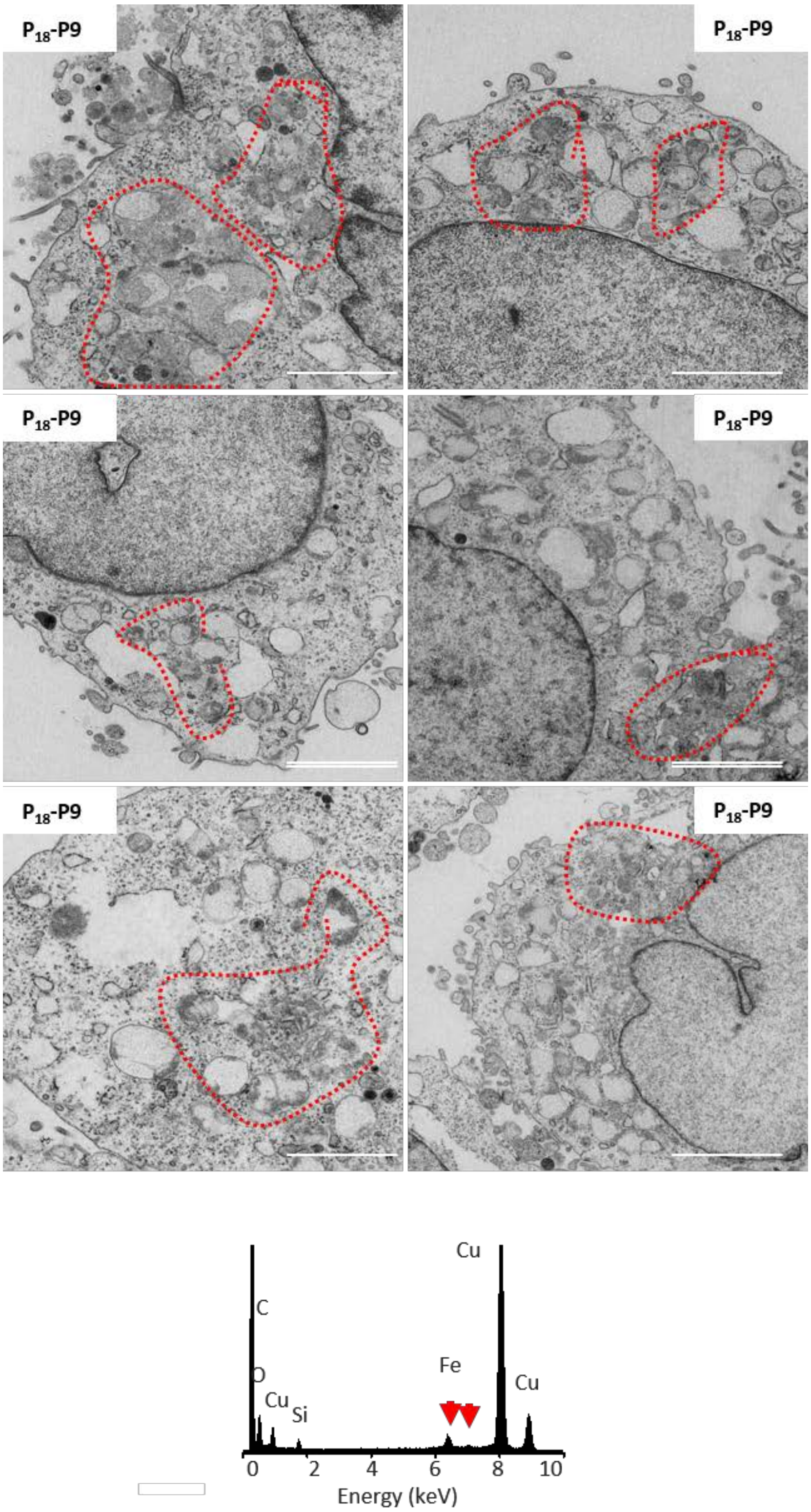

Supplementary Figure 46. The TEM images of HeLa cell ultrathin sections after treatment with P_{18} labelled peptide 9 and the red dot circle indicates the gel formed by **P_{18}-P9** and the EDS scale region. *Scale bar*, 2 μm.

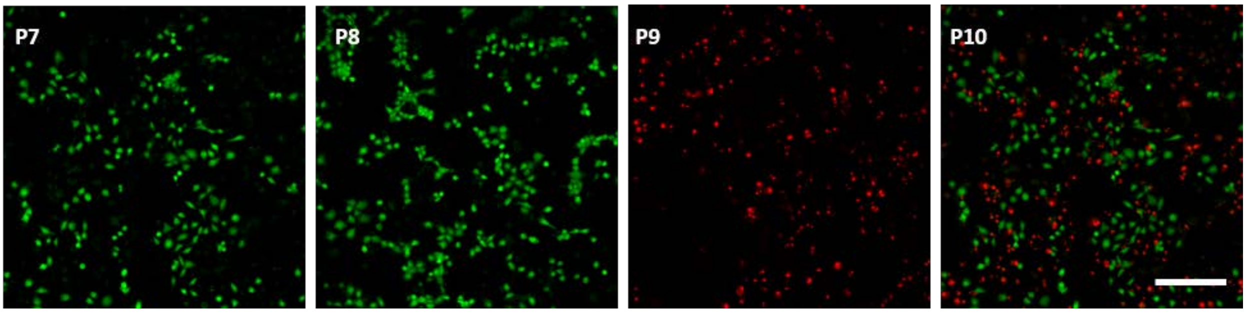

Supplementary Figure 47. Live/dead assays of peptide 7, 8, 9 and 10 (250 μM, 12h) treated HeLa cells. *Scale bar*, 200 μm.

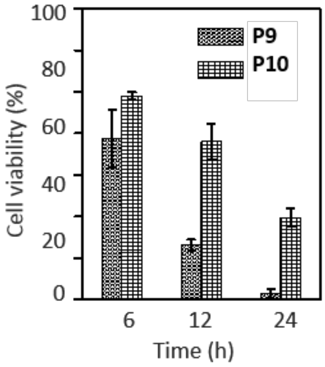

Supplementary Figure 48. Time-dependent cell viability of HeLa cells after treated by peptide 9 and 10 with a dose of 600 μM. *Error bars* are standard deviation and obtained from triplicated experiments.

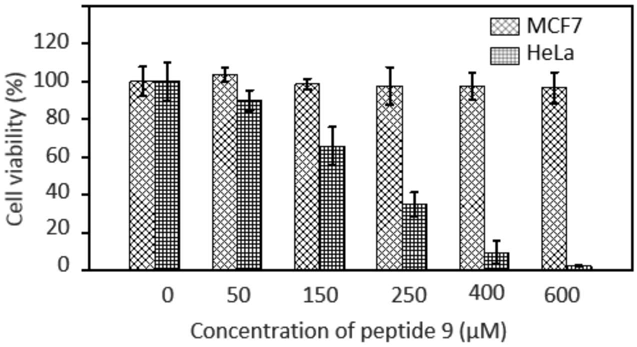

Supplementary Figure 49. Concentration effect of peptide 9 on cell viabilities of HeLa and MCF7 for 24 h. *Error bars* are standard deviation and obtained from triplicated experiments.

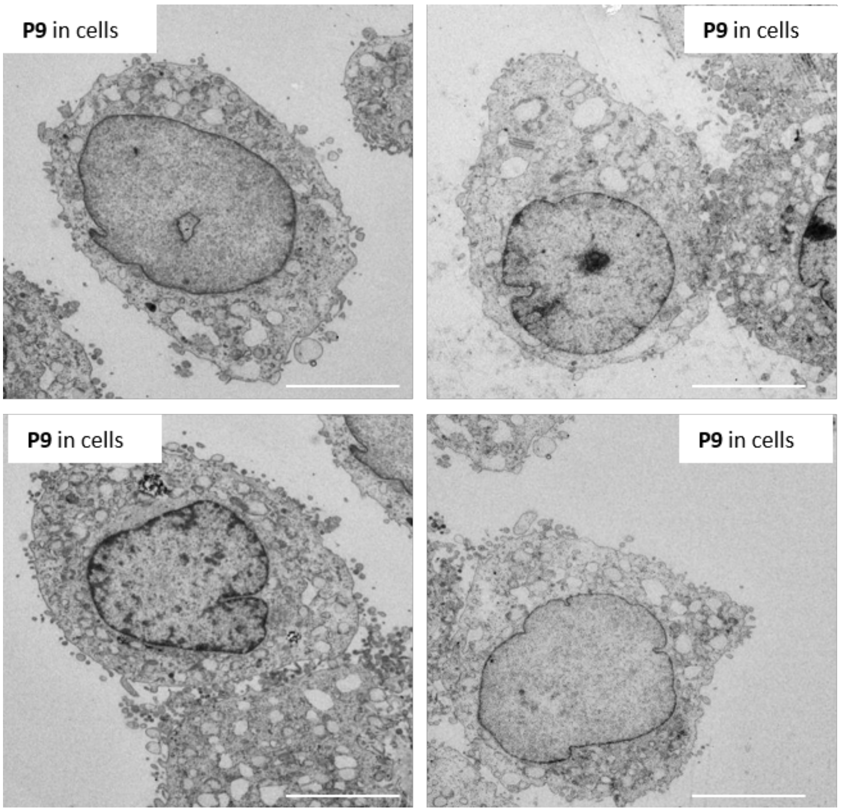

Supplementary Figure 50. The TEM images of ultrathin sections of peptide 9 treated HeLa cells. *Scale bar*, 5 µm.

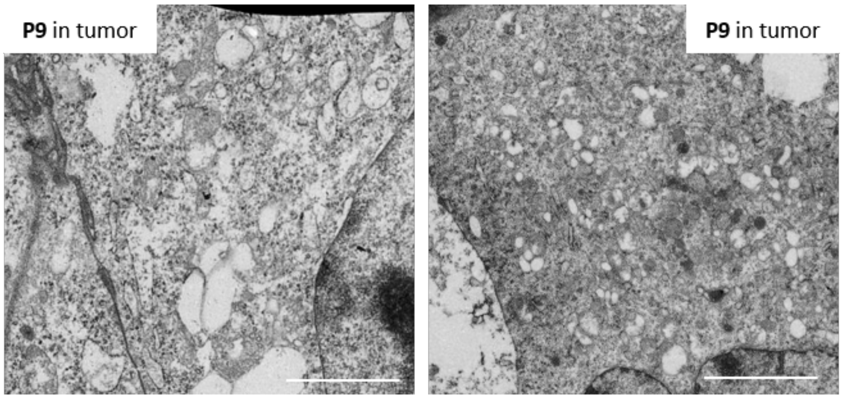

Supplementary Figure 51. The TEM images of ultrathin sections of peptide 9 treated tumor tissues. *Scale bar*, 2 µm.

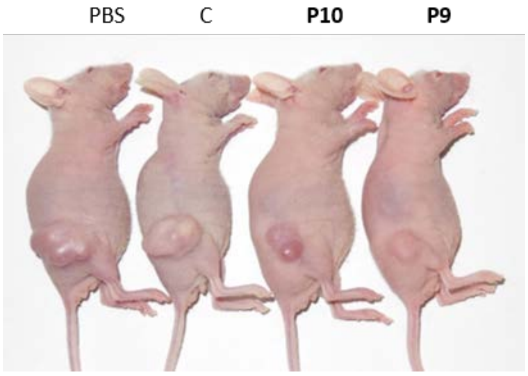

Supplementary Figure 52. Photograph of xenografts-bearing mice after treatment by PBS, peptide control (C), peptide 7(**P7**) and 10 (**P10**) for two weeks.

Uncropped images of blots in Figure 3e

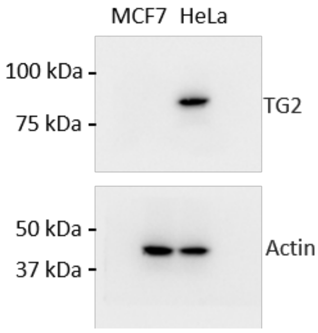

Supplementary Figure 53

Uncropped images of blots in Figure 3g

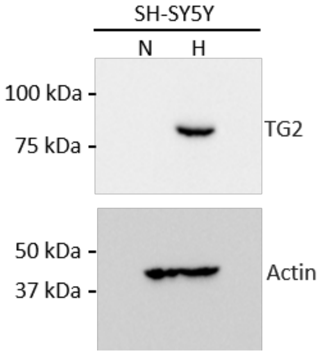

Supplementary Figure 54

Supplementary Table 1. Optimization of the substrate to TG2 ratio

Substrate(peptide 4)/TGase (TG2)(μM U^{-1})	Polypeptide (MW)	PDI (Mw/Mn)	Number of repeat unit
10/1	12600	1.12	12-13
30/1	19000	1.31	17-18
50/1	26600	1.21	27-28

Supplementary Methods

General information

Piperidine,4-methylmorpholine (NMM),*O*-(benzotriazol-1-yl)-N,N,N′,N′-tetramethyluronium hexafluorophosphate (HBTU), ninhydrin, phenol, ascorbic acid (VC), Triisopropylsilane (TIPS), trifluoroacetic acid (TFA), 2,5-dihydroxybenzoic acid (DHB), Transglutaminase and N-{2-[(7-N,N-dimethylaminosulfonyl)-2,1,3-benzoxadiazol-4-yl](methyl)amino}ethyl-N-methylacrylamide(DBD) were purchased from Sigma-Aldrich Co.. Dulbecco's Modification of Eagle Medium (DMEM), fetal bovine serum (FBS) and trypsin were obtained from Hyclone, Logan, UT. Inertsil C18 HPLC column (ODS-3, 5 µm, 4.6 × 250 mm) for peptide analysis was purchased from Shimadzu Corporation. The HPLC grade solvents methanol and acetonitrile were purchased from Aldrich, Fisher Scientific. Fmoc-Lys(OtBu)-Wang resin and amino acids such as Fmoc-Gly-OH, Fmoc-Asp(OtBu)-OH, Fmoc-Gln(Trt)-OH, Fmoc-Pro-OH, Fmoc-Val-OH, Fmoc-Phe-OH, Fmoc-Leu-OH, Fmoc-Arg-OH and Fmoc-Ala-OH for peptide synthesis were obtained from GL Biochem (Shanghai) Ltd. 96-well coning culture plates were purchased from Coning Company. All the other solvents used in the research were purchased from Sinopharm Chemical Reagent Beijing Co., Ltd or Beijing (China) Chemical Company. Fluorescence spectra and UV-vis spectra were recorded using an F-280 fluorescence spectrophotometer and a Shimadzu 3600 UV-vis spectrophotometer respectively. The morphology of the aggregates was observed by NOVA Nano SEM 430 scanning electron microscope. The HPLC was carried out by Waters 626 system. Mass spectra were obtained by Microflex LRF MALDI-TOF with a matrix of DHB (20 mg mL^{-1}) in TA30 (H_2O 70%, acetonitrile 30%, 0.1% TFA, v/v solution) by the dried droplet method on the AnchorChip of a MSP 96 polished plate. Cell fluorescence microscopic observations were recorded using Zeiss 710 single photon confocal microscope.

Methods

Peptide synthesis, labelling and characterization. All peptides were synthesized by solid-phase methods using a standard Fmoc-chemistry. The 0.3 mmol scale protocol with C-terminal capping protection strategy by amide was used. The deprotection of Fmoc group on N-terminal was used piperidine (20% v/v) in anhydrous DMF. Qualitative Fmoc deprotection was confirmed by a ninhydrin test (ninhydrin, phenol, VC 1:1:1 v/v). Amino acid activation was achieved by NMM (0.4 M) and HBTU (the same mole of amino acid) in anhydrous DMF. The signal molecules of P_{18} and DBD were treated similar to amino acids in the last peptide coupling step, while, FITC and Cy5 were coupled without NMM and HBTU addition. Finally, the cleavage from the resin and deprotection of the amino acid side chains were performed by

reaction with the mixture of TFA (95% v/v), H_2O (2.5% v/v) and TIPS (2.5%, v/v) for 30 min in ice bath and then at room temperature for 2.5 h. After separated with the resin, the mixture above was vacuum rotary evaporated to remove the TFA. The peptides were then precipitated in cold anhydrous diethyl ether, collected by centrifuge and dried under vacuum without further purification. Peptides were analyzed by analytical HPLC chromatograms (with a dual λ absorbance detector, a C18 column and a linear gradient of acetonitrile/water with 0.1% TFA respectively from 5%/95% to 60%/40% and a flow speed of 0.5 mL min^{-1}) and MALDI-TOF mass spectra (with the matrix of saturated DHB in TA30 by dried droplet method on the AnchorChip of MSP 96 polished).

Characterization of linear grown ELPs. Molecular weights (MW) and polydispersity indexes (PDI) of the synthesized polypeptides were measured by GPC (Shimadzu) with a Shimadzu RID-20A refractive index detector. The MW and PDI were analysed under a condition of 40 ^{o}C in DMF (0.4% LiBr) with a flow rate of 1.0 ml min^{-1} and calculated based on universal calibration method using polystyrene standards.

Characterization of 3D gel-like ELPs. To get the minimum gelation concentration, concentration dependent ligation experiments were studied by inverted vial method. In a typical experiment, four different concentrations of peptide 9 (*i.e.*, 70, 150, 250 and 400 μM) were vortexed with TG2 at 37 °C. Gelation time varied from 0.5 to 6 h depending on monomer concentrations; gels formed faster when higher monomer peptide concentrations were used. We found the minimum gelation concentration was about 70 μM. Rheological experiments were carried out with a US 200 rheometer (Anton Paar) using a conical plate (25 mm diameter) configuration at 37 ^{o}C. In typical examples, 0.5 mL of peptide 9 or 10 stock solution (5 wt%, in HEPES buffer solution) and 0.5 mL of TG2 stock solution (20 U ml^{-1}, in HEPES buffer solution) were mixed. The substrate/TG2 solution was then applied to the rheometer, and the upper plate was immediately lowered to a measuring gap size of 0.5 mm, and the measurement was started. To prevent evaporation, mineral oil was used at the edge of the samples. The evolution of the storage modulus (G') and loss modulus (G'') was recorded as a function of time and for a period of 70 min. A frequency of 0.5 Hz and a strain of 5% were applied in order to maintain the linear viscoelastic regime. The crossover point of G' and G'' was considered to be the gelation point. The crosslinking efficiency CE (%) of **P9** and **P10** was indirectly calculated from the soluble fraction measurements, *i.e.*, from the ratio of the dry weight of **P9** after the purification process (removing of peptide monomers, TG2 and water soluble monomers) to the dry hydrogel weight immediately after synthesis, according to the Eq.1:

$$CE\ (\%) = \frac{\mathrm{Wpd}}{\mathrm{Wid}} \times 100 \qquad (1)$$

Where Wpd is the purified dry weight of polypeptide and Wid is the initial dry weight of peptide monomers input after the TG2 catalyzed synthesis before the purification.

Differential scanning calorimetry (DSC). The thermosensitivity of the peptide monomers (4, 7 and 9) or polypeptides (**P4**, **P7**-**P10**) were determined using differential scanning calorimeter (Diamond DSC, PerkinElmer, America). The samples were immersed in DI water at 4 ^{o}C and allowed to reach the equilibrium state. ~ 8 mg of samples were placed in a hermetic sample pan and sealed for DSC analysis. The scanning was at a temperature ramp rate of 2 ^{o}C min^{-1} under nitrogen flow.

Confocal imaging. The HeLa, MCF-7 and SH-SY5Y cells were seeded into confocal dish with a density of 20,000 cells per well and further incubated for 24 h before introducing to peptide monomers. After washed by PBS, a total of 2 ml of peptide monomers with a concentration of 600 μM in medium was added for 12 h. Then, the cells were stained by Hoechst and Dil for labeling the nucleus and membrane respectively. The **P7**-**P10** images were obtained at 37 ^{o}C. The extended state and collapsed state of **P4** were obtained under a temperature condition of 37 ^{o}C and 4 ^{o}C, respectively.

Scanning electron microscope (SEM). Polypeptides in aqueous solution were dropped on silica wafers (cleaned by plasma). Then, the extended state of **P4** and collapsed state of **P7** and **P9** were dried at 37 ^{o}C under vacuum condition. Besides, the collapsed state of **P4** was prepared on low temperature chamber with ice under vacuum to dry. SEM images were acquired on Tecnai G2 F20 U-TWIN under an acceleration voltage of 10.0 kV, spot size of 3.0, and a working distance of 5.0 mm.

Transmission electron microscopy (TEM). Peptides treated cells or tumour tissues were harvested and fixed with 2% formaldehyde and 2.5% glutaraldehyde in PBS buffer overnight. After washing with PB buffer for 10 min per time for triple times, the cells were fixed with 1% osmium containing PB buffer for 2 h at room temperature. Subsequently, samples were washed several times with PB buffer similar with the protocol above. Samples were then dehydrated with a graded series of ethanol (50, 70, 80, 90, 95, 100%) on ice for 30 min for each step. Samples were infiltrated with a graded of series of mixtures (ethanol/EPON 812 resin: 2/1, 1/1, 1/2) at RT for 1h for each step. Pure resin was added the next day on ice, changed and left for overnight. Finally, samples were placed into gelatine capsules and filled with pure EPON 812 resin at 37 ^{o}C and 45 ^{o}C for 24 h, respectively. EPON 812 resin was polymerized for 48 h at 60 ^{o}C. Ultrathin sections were cut with a diamond knife and sections picked up with formvar-coated copper grids (300 mesh). Counter-staining of the sections was performed with osmic acid (1%) for 1 h and uranyl acetate (4%) for 15 min, respectively. After air-drying samples were examined in HITACHI HT7700 transmission electron microscope at an acceleration voltage of 80 kV. The energy dispersive analysis was carried out by Tecnai G2 F20 U-TWIN electron microscope.

CCK-8 assay. HeLa cells were seeded into 96-well plates at 7000 cells per well and then incubated for 24 h at 37 ^oC under 5% CO_2. For cytotoxicity assay, a total of 200 µl of testing materials (peptide monomers) with various concentrations (0-1 mM) in DMEM was added and the cells were incubated for 24 h. After sufficient PBS washing, CCK-8 assays were used to determine the cell ability. The absorbance of each well was measured at 450 nm with a plate reader.

Long-term retention. The HeLa and MCF-7 cells were seeded into confocal dish with a density of 50,000 cells per well. A total of 2 ml of FITC labeled control peptide, peptide 7 and 8 with a concentration of 600 µM in medium was added. At the time interval at 0.5, 4, 12 and 24 h, the cells were washed by PBS twice to remove the peptide in the medium. Then, the cells were stained by Hoechst and Dil for labeling the nucleus and membrane respectively. The confocal images were obtained at a temperature at 37 ^oC.

***In vivo* bioimaging experiments.** Animal experiments were carried out complying with NIH guidelines for the care and euthanasia of laboratory animals of National Center for Nanoscience and Technology Animal Study Committee's requirement and according to the protocol approved by the Institutional Animal Care. 2000,000 HeLa cells, collected in PBS (200 µl, pH 7.4), were subcutaneously (*s.c.*) injected into the right lateral hind hip of the female BALB/c nude mice. For *in vivo* bioimaging, the PBS and Cy 5.5 labelled peptides (control, 7 and 8) were separately introduced to mice with a dose of 5.0 mg kg^{-1}. The whole-body fluorescence images were monitored at 0h, 1h, 4h, 8h, 12h and 24h using a Maestro fluorescence imager.
